# The P323L substitution in the SARS-CoV-2 polymerase (NSP12) confers a selective advantage during infection

**DOI:** 10.1186/s13059-023-02881-5

**Published:** 2023-03-13

**Authors:** Hannah Goldswain, Xiaofeng Dong, Rebekah Penrice-Randal, Muhannad Alruwaili, Ghada T. Shawli, Tessa Prince, Maia Kavanagh Williamson, Jayna Raghwani, Nadine Randle, Benjamin Jones, I’ah Donovan-Banfield, Francisco J. Salguero, Julia A. Tree, Yper Hall, Catherine Hartley, Maximilian Erdmann, James Bazire, Tuksin Jearanaiwitayakul, Malcolm G. Semple, Peter J. M. Openshaw, J. Kenneth Baillie, ISARIC4C Investigators, J. Kenneth Baillie, Malcolm G. Semple, Peter J. M. Openshaw, Gail Carson, Beatrice Alex, Petros Andrikopoulos, Benjamin Bach, Wendy S. Barclay, Debby Bogaert, Meera Chand, Kanta Chechi, Graham S. Cooke, Ana da Silva Filipe, Thushan de Silva, Annemarie B. Docherty, Gonçalo dos Santos Correia, Marc-Emmanuel Dumas, Jake Dunning, Tom Fletcher, Christoper A. Green, William Greenhalf, Julian L. Griffin, Rishi K. Gupta, Ewen M. Harrison, Julian A. Hiscox, Antonia Ying Wai Ho, Peter W. Horby, Samreen Ijaz, Saye Khoo, Paul Klenerman, Andrew Law, Matthew R. Lewis, Sonia Liggi, Wei Shen Lim, Lynn Maslen, Alexander J. Mentzer, Laura Merson, Alison M. Meynert, Shona C. Moore, Mahdad Noursadeghi, Michael Olanipekun, Anthonia Osagie, Massimo Palmarini, Carlo Palmieri, William A. Paxton, Georgios Pollakis, Nicholas Price, Andrew Rambaut, David L. Robertson, Clark D. Russell, Vanessa Sancho-Shimizu, Caroline J. Sands, Janet T. Scott, Louise Sigfrid, Tom Solomon, Shiranee Sriskandan, David Stuart, Charlotte Summers, Olivia V. Swann, Zoltan Takats, Panteleimon Takis, Richard S. Tedder, A. A. Roger Thompson, Emma C. Thomson, Ryan S. Thwaites, Lance C. W. Turtle, Maria Zambon, Hayley Hardwick, Chloe Donohue, Fiona Griffiths, Wilna Oosthuyzen, Cara Donegan, Rebecca G. Spencer, Lisa Norman, Riinu Pius, Thomas M. Drake, Cameron J. Fairfield, Stephen R. Knight, Kenneth A. Mclean, Derek Murphy, Catherine A. Shaw, Jo Dalton, Michelle Girvan, Egle Saviciute, Stephanie Roberts, Janet Harrison, Laura Marsh, Marie Connor, Sophie Halpin, Clare Jackson, Carrol Gamble, Daniel Plotkin, James Lee, Gary Leeming, Andrew Law, Murray Wham, Sara Clohisey, Ross Hendry, James Scott-Brown, Victoria Shaw, Sarah E. McDonald, Seán Keating, Katie A. Ahmed, Jane A. Armstrong, Milton Ashworth, Innocent G. Asiimwe, Siddharth Bakshi, Samantha L. Barlow, Laura Booth, Benjamin Brennan, Katie Bullock, Benjamin W. A. Catterall, Jordan J. Clark, Emily A. Clarke, Sarah Cole, Louise Cooper, Helen Cox, Christopher Davis, Oslem Dincarslan, Chris Dunn, Philip Dyer, Angela Elliott, Anthony Evans, Lorna Finch, Lewis W. S. Fisher, Terry Foster, Isabel Garcia-Dorival, Philip Gunning, Rebecca L. Jensen, Christopher B. Jones, Trevor R. Jones, Shadia Khandaker, Katharine King, Robyn T. Kiy, Chrysa Koukorava, Annette Lake, Suzannah Lant, Diane Latawiec, Lara Lavelle-Langham, Daniella Lefteri, Lauren Lett, Lucia A. Livoti, Maria Mancini, Sarah McDonald, Laurence McEvoy, John McLauchlan, Soeren Metelmann, Nahida S. Miah, Joanna Middleton, Joyce Mitchell, Shona C. Moore, Ellen G. Murphy, Jack Pilgrim, Will Reynolds, P. Matthew Ridley, Debby Sales, Victoria E. Shaw, Rebecca K. Shears, Benjamin Small, Krishanthi S. Subramaniam, Agnieska Szemiel, Aislynn Taggart, Jolanta Tanianis-Hughes, Jordan Thomas, Erwan Trochu, Libby van Tonder, Eve Wilcock, J. Eunice Zhang, Lisa Flaherty, Nicole Maziere, Emily Cass, Alejandra Doce Carracedo, Nicola Carlucci, Anthony Holmes, Hannah Massey, Lee Murphy, Sarah McCafferty, Richard Clark, Angie Fawkes, Kirstie Morrice, Alan Maclean, Nicola Wrobel, Lorna Donnelly, Audrey Coutts, Katarzyna Hafezi, Louise MacGillivray, Tammy Gilchrist, Kayode Adeniji, Daniel Agranoff, Ken Agwuh, Dhiraj Ail, Erin L. Aldera, Ana Alegria, Sam Allen, Brian Angus, Abdul Ashish, Dougal Atkinson, Shahedal Bari, Gavin Barlow, Stella Barnass, Nicholas Barrett, Christopher Bassford, Sneha Basude, David Baxter, Michael Beadsworth, Jolanta Bernatoniene, John Berridge, Colin Berry, Nicola Best, Pieter Bothma, David Chadwick, Robin Brittain-Long, Naomi Bulteel, Tom Burden, Andrew Burtenshaw, Vikki Caruth, David Chadwick, Duncan Chambler, Nigel Chee, Jenny Child, Srikanth Chukkambotla, Tom Clark, Paul Collini, Catherine Cosgrove, Jason Cupitt, Maria-Teresa Cutino-Moguel, Paul Dark, Chris Dawson, Samir Dervisevic, Phil Donnison, Sam Douthwaite, Andrew Drummond, Ingrid DuRand, Ahilanadan Dushianthan, Tristan Dyer, Cariad Evans, Chi Eziefula, Chrisopher Fegan, Adam Finn, Duncan Fullerton, Sanjeev Garg, Sanjeev Garg, Atul Garg, Effrossyni Gkrania-Klotsas, Jo Godden, Arthur Goldsmith, Clive Graham, Elaine Hardy, Stuart Hartshorn, Daniel Harvey, Peter Havalda, Daniel B. Hawcutt, Maria Hobrok, Luke Hodgson, Anil Hormis, Michael Jacobs, Susan Jain, Paul Jennings, Agilan Kaliappan, Vidya Kasipandian, Stephen Kegg, Michael Kelsey, Jason Kendall, Caroline Kerrison, Ian Kerslake, Oliver Koch, Gouri Koduri, George Koshy, Shondipon Laha, Steven Laird, Susan Larkin, Tamas Leiner, Patrick Lillie, James Limb, Vanessa Linnett, Jeff Little, Mark Lyttle, Michael MacMahon, Emily MacNaughton, Ravish Mankregod, Huw Masson, Elijah Matovu, Katherine McCullough, Ruth McEwen, Manjula Meda, Gary Mills, Jane Minton, Mariyam Mirfenderesky, Kavya Mohandas, Quen Mok, James Moon, Elinoor Moore, Patrick Morgan, Craig Morris, Katherine Mortimore, Samuel Moses, Mbiye Mpenge, Rohinton Mulla, Michael Murphy, Megan Nagel, Thapas Nagarajan, Mark Nelson, Lillian Norris, Matthew K. O’Shea, Igor Otahal, Marlies Ostermann, Mark Pais, Carlo Palmieri, Selva Panchatsharam, Danai Papakonstantinou, Hassan Paraiso, Brij Patel, Natalie Pattison, Justin Pepperell, Mark Peters, Mandeep Phull, Stefania Pintus, Jagtur Singh Pooni, Tim Planche, Frank Post, David Price, Rachel Prout, Nikolas Rae, Henrik Reschreiter, Tim Reynolds, Neil Richardson, Mark Roberts, Devender Roberts, Alistair Rose, Guy Rousseau, Bobby Ruge, Brendan Ryan, Taranprit Saluja, Matthias L. Schmid, Aarti Shah, Prad Shanmuga, Anil Sharma, Anna Shawcross, Jeremy Sizer, Manu Shankar-Hari, Richard Smith, Catherine Snelson, Nick Spittle, Nikki Staines, Tom Stambach, Richard Stewart, Pradeep Subudhi, Tamas Szakmany, Kate Tatham, Jo Thomas, Chris Thompson, Robert Thompson, Ascanio Tridente, Darell Tupper-Carey, Mary Twagira, Nick Vallotton, Rama Vancheeswaran, Lisa Vincent-Smith, Shico Visuvanathan, Alan Vuylsteke, Sam Waddy, Rachel Wake, Andrew Walden, Ingeborg Welters, Tony Whitehouse, Paul Whittaker, Ashley Whittington, Padmasayee Papineni, Meme Wijesinghe, Martin Williams, Lawrence Wilson, Sarah Cole, Stephen Winchester, Martin Wiselka, Adam Wolverson, Daniel G. Wootton, Andrew Workman, Bryan Yates, Peter Young, Stevan R. Emmett, Paul Digard, David A. Matthews, Lance Turtle, Alistair C. Darby, Andrew D. Davidson, Miles W. Carroll, Julian A. Hiscox

**Affiliations:** 1 Institute of Infection, Veterinary and Ecological Sciences, University of Liverpool, Liverpool, UK; 2 Medical Laboratory Technology Department, Northern Border University, Arar City, Saudi Arabia; 3 School of Cellular and Molecular Medicine, University of Bristol, Bristol, UK; 4 Department of Zoology, University of Oxford, Oxford, UK; 5 NIHR Health Protection Unit in Emerging and Zoonotic Infections, Liverpool, UK; 6 UK Health Security Agency, Salisbury, UK; 7 Department of Microbiology, Mahidol University, Salaya, Thailand; 8 Department of Respiratory Medicine, Alder Hey Children’s Hospital, Liverpool, UK; 9 National Heart and Lung Institute, Imperial College London, London, UK; 10 The Roslin Institute, University of Edinburgh, Edinburgh, UK; 11 Royal United Hospitals Bath NHS Foundation Trust, Bath, UK; 12 Bristol Medical School, University of Bristol, Bristol, UK; 13 Nuffield Department of Medicine, University of Oxford, Oxford, UK; 14 A*STAR Infectious Diseases Laboratories (A*STAR ID Labs), Agency for Science, Technology and Research (A*STAR), Singapore, Singapore

**Keywords:** SARS-CoV-2, Evolution, Selection, Spike protein, Polymerase, NSP12, COVID-19, P323L

## Abstract

**Background:**

The mutational landscape of SARS-CoV-2 varies at the dominant viral genome sequence and minor genomic variant population. During the COVID-19 pandemic, an early substitution in the genome was the D614G change in the spike protein, associated with an increase in transmissibility. Genomes with D614G are accompanied by a P323L substitution in the viral polymerase (NSP12). However, P323L is not thought to be under strong selective pressure.

**Results:**

Investigation of P323L/D614G substitutions in the population shows rapid emergence during the containment phase and early surge phase during the first wave. These substitutions emerge from minor genomic variants which become dominant viral genome sequence. This is investigated in vivo and in vitro using SARS-CoV-2 with P323 and D614 in the dominant genome sequence and L323 and G614 in the minor variant population. During infection, there is rapid selection of L323 into the dominant viral genome sequence but not G614. Reverse genetics is used to create two viruses (either P323 or L323) with the same genetic background. L323 shows greater abundance of viral RNA and proteins and a smaller plaque morphology than P323.

**Conclusions:**

These data suggest that P323L is an important contribution in the emergence of variants with transmission advantages. Sequence analysis of viral populations suggests it may be possible to predict the emergence of a new variant based on tracking the frequency of minor variant genomes. The ability to predict an emerging variant of SARS-CoV-2 in the global landscape may aid in the evaluation of medical countermeasures and non-pharmaceutical interventions.

**Supplementary Information:**

The online version contains supplementary material available at 10.1186/s13059-023-02881-5.

## Background

There are distinct lineages of SARS-CoV-2 currently circulating worldwide and many more have been displaced [1]. The genome of SARS-CoV-2 is changing as the pandemic continues. Replication and transcription of the SARS-CoV-2 genome directly drives three types of genetic change in the virus. The first is recombination, which is a natural consequence of subgenomic messenger RNA (sgmRNA) synthesis. This process may account for insertions and deletions, for example, observed in and around the furin cleavage site in the spike glycoprotein [2] and other genes [3]. The second driver of genetic change is the continual accruing of point mutations. These changes may confer advantages in transmission, such as A23403G, encoding the D614G substitution in the spike protein [4], which has come to predominate in global SARS-CoV-2 sequences since the start of the outbreak [5]. Such point mutations may be driven by errors during RNA synthesis by the viral encoded RNA-dependent RNA polymerase (NSP12) and larger replication complex, and/or by host-mediated processes [6, 7]. The third mechanism is the potential generation and selection of new transcription regulatory signals (TRSs) and the synthesis of new viral sgmRNAs and proteins [8]. Promiscuous recombination and mutation in coronaviruses may allow these viruses to overcome selection pressures, transit population bottlenecks, and result in the emergence of new variants [9, 10].

Diversity in dominant and minor viral genomic variants exists in humans/animals infected with SARS-CoV-2 [10]. These genomes will have both synonymous (non-coding) and non-synonymous (coding) differences. Minor genomic variations may be selected for when they confer a selective advantage when the virus enters a new host. This has been demonstrated with the adaptation of Ebola virus in a guinea pig model of infection [11]. Alternatively, the variation may exist at a minor variant level but nevertheless impact upon virus biology, for example, with the Ebola virus RNA-dependent RNA polymerase (L protein) and the relationship with overall viral load in patients with Ebola virus disease [12].

Since the start of the COVID-19 pandemic, different dominant viral genome sequences and non-synonymous changes appear to rise and fall in the SARS-CoV-2 global sequences [1]. The D614G spike protein variant of SARS-CoV-2 was first detected in China, in January 2020, and by May 2020 approximately 80% of viruses sequenced globally contained this substitution. The major clade containing D614G (Pango lineages B.1 and sub-lineages) contained potentially linked substitutions, including C14408U in NSP12 that confers a P323L substitution. However, lineages such as A.19 and A.2.4, gained D614G in the spike protein but not P323L in NSP12 [13], which may have contributed to their lower global prevalence. Therefore, whether P323L in NSP12 conferred a fitness advantage in the context of the D614G substitution and was subject to selection pressure is unknown.

To investigate the intra-host selection pressure for the P323L variant, sequential samples from patients with COVID-19 prior to and during the D614G/P323L change in the UK were sequenced to study both the dominant viral genome sequence and minor variant genomes. Additionally, a lineage B SARS-CoV-2 with L323 and G614 in the minor variant population was used to infect two non-human primate (NHP) models [13], cynomolgus (*Macaca fascicularis*), and rhesus (*Macaca mulatta*) macaques. Longitudinal sampling indicated that L323 became part of the dominant viral genome sequence, but not G614. Reverse genetics analysis of P323L in the background of a G614 virus indicated that the L323 variant showed greater abundance of viral RNA and proteins and a smaller plaque morphology compared to P323. Overall, the P323L change provided an additive advantage to variants with D614G in the spike protein. In the wider context, the work indicated that an emerging dominant sequence could be predicted by analysis of minor variant genomes.

## Results

### Identification of the emergence of a P323L substitution in NSP12 in SARS-CoV-2 RNA in a patient during infection

**Fig. 1.**
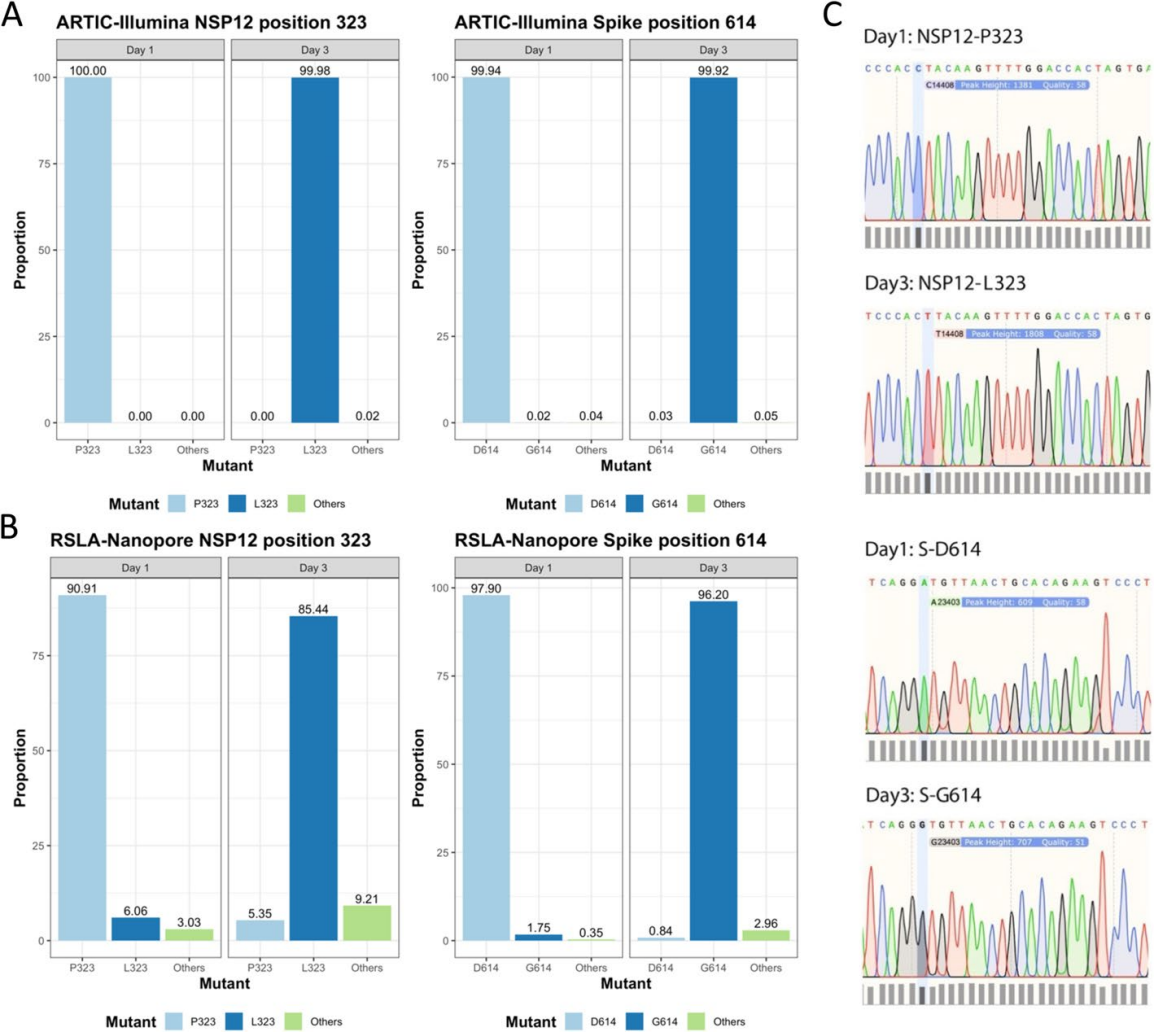
Sequence analysis and amino acid substitution in NSP12 (P323L) and the spike protein (D614G) between the first and third days of sampling in a patient hospitalized with COVID-19. Three different sequencing approaches were used: **A** an ARTIC-Illumina approach and **B** a RSLA-Nanopore approach to show the amino acid variation frequencies of NSP12 (at codon position 323) and spike protein (codon position 614). **C** Sanger sequence analysis of the amplicons used to investigate the change of dominant viral genome sequence around the sites within NSP12 (codon position 323) and spike protein (codon position 614). These samples were gathered under the auspices of ISARIC 4C

To identify whether the P323L substitution occurred rapidly in NSP12, nasopharyngeal swabs were identified in the ISARIC 4C biobank that were obtained from patients infected with lineage B SARS-CoV-2 prior to the major shift from P323 to L323 and D614 to G614 in the UK. Samples were further down selected based on clinical information providing dates of symptom onset, first sample, and subsequent longitudinal samples. This identified 12 patients with longitudinal samples from a total of 472 patients. RNA was isolated from the longitudinal swabs and used as a template for the amplification of SARS-CoV-2 genome and sgmRNAs using both short- (ARTIC-Illumina) and longer-read length (Rapid Sequencing of Long Amplicons-Nanopore (RSLA-Nanopore)) sequencing [14, 15]. These longitudinal samples had sufficient read depth to call a consensus for the dominant viral genome sequence and to derive information on the frequency of minor genomic variants, focusing on codons 323 in NSP12 and 614 in the spike protein. In one patient, who was isolated in the intensive care unit at the Royal Liverpool Hospital, both sequencing approaches indicated that the P323L and D614G substitutions occurred in the dominant SARS-CoV-2 genome between the first and second samples, taken 2 days apart (Fig. 1A, B, respectively). To independently confirm this observation, the source RNA was Sanger sequenced with primers to generate longer amplicons around the potential substitution sites. The data validated that for NSP12 the codon encoding the amino acid at position 323 changed from CCU (encoding P) to CUU (encoding L) (Fig. 1C). For the spike protein, the codon encoding the amino acid at position 614 changed from GAU (encoding D) to GGU (encoding G) (Fig. 1C). Therefore, the data suggested that both P323L and D614G were rapidly selected in the patient over a 2-to-3-day period. Another possibility is that the patient was infected with a P323/D614 variant and subsequently became infected with an L323/G614 variant through nosocomial infection in the hospital setting. However, we consider this possibility unlikely as this patient was isolated in the intensive care unit of Liverpool University Hospitals. There were relatively few other COVID-19 patients in the hospital at that period of the containment phase, although the contribution of asymptomatic transmission was not widely recognized.

**Fig. 2.**
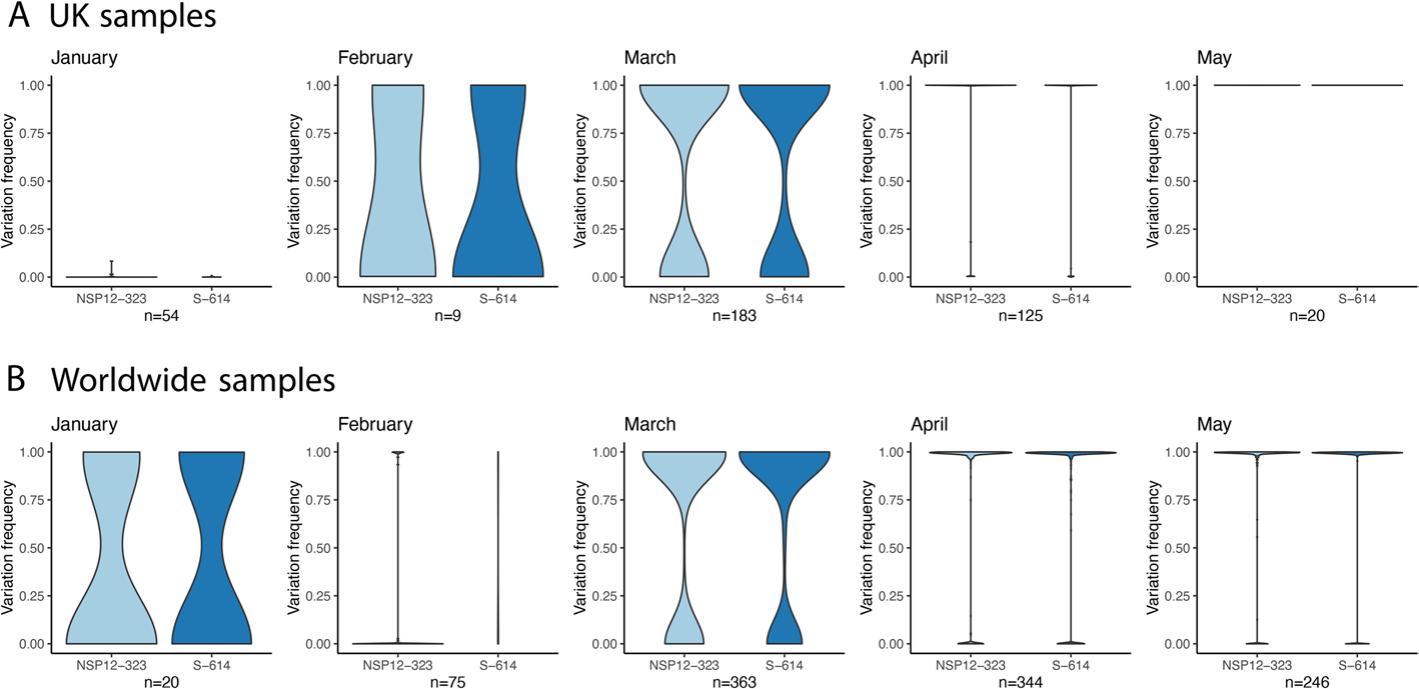
Analysis of the ratio of P323:L323 (light blue) and D614:G614 (blue) in 377 patient samples between January 2020 and May 2020 in the **A** UK and **B** worldwide (a ratio of 1.00= L323/G614 dominant and the violin plot indicates the number of samples). SARS-CoV-2 sequence was obtained from nasopharyngeal swabs from 377 hospitalized patients. The width of the violin plot indicates the number of samples/patients with the frequency on the *y*-axis. The data shows the transition from P323L and D614G over time in the minor variant genomes, such that by April 2020 in the UK, the L323 and G614 substitutions were part of the dominant viral genome sequence and by May 2020, there was no evidence of P323 and D614 at the dominant level. The *y*-axis (variation frequency) is in the direction of P323 to L323 and D614 to G614, such that a viral population with 100% L323 or G614 would be shown with a variation frequency of 1.00. Likewise, if there is a variation frequency of 0.00, this would mean that there was a viral population with 100% P323 or D614

The distribution of P323L and D614G at the minor genomic variant level was evaluated in the human population between January 2020 and June 2020, when these substitutions became part of the dominant viral genome sequence. This included publicly available sequence data for the UK for January 2020 and bespoke sequencing of samples gathered under the auspices of ISARIC 4C for February to May 2020. For the latter, SARS-CoV-2 was sequenced from nasopharyngeal swabs sampled from 522 patients in the UK over that period and usable data obtained from 377 (Fig. 2). For analysis of worldwide samples, this made use of publicly available sequence data.

The data (Fig. 2) indicated that there was increasing prevalence from P323 to L323 and D614 to G614 in the February to March sampling period in the UK. For both February and March 2020, viruses with P323, L323, D614, and G614 at the dominant viral genome level were co-circulating in the human population. However, by May 2020, all the dominant viral genome sequences from this cohort contained L323 and G614, suggesting either strong selection pressure and/or multiple founder effects. Worldwide diversity was observed at these positions in January 2020, then followed a similar pattern to the UK with the major transition to L323 and G614 in April/May 2020.

### Longitudinal analysis of variation in NHPs and cell culture

To investigate whether the P323L substitution was driven by strong selection pressure, nasal washes were taken daily from cynomolgus and rhesus macaques (12 animals of each species, a 50:50 mix of males and females [16]). These had been infected via the intratracheal route with 5 × 10^6^ pfu of SARS-CoV-2 (Victoria/01/2020) [16]. This stock contained P323 and D614 at the dominant viral genome level [16]. The isolate had been passaged three times in cell culture to generate stock virus prior to infection of the cynomolgus and rhesus macaques. Sequencing of the stock virus indicated a very low proportion of NSP12 L323 and spike G614 at the minor variant level (Table 1).

**Table 1:** Amino acid coverage at positions NSP12-323 and spike-614 in the SARS-CoV-2 Victoria/01/2020 stock

Protein	Amino acid position	Read coverage	Variant	Number of reads	Proportion
NSP12	323	33629	P	33,423	0.9939
			L	10	0.0003
			others	196	0.0058
Spike	614	41047	D	40,901	0.9964
			G	7	0.0002
			others	139	0.0034

The number of reads per P, L, and other amino acids and per D, G, and other amino acids at positions 323 in NSP12 and 614 in the spike protein respectively were determined by ARTIC-Illumina sequencing of the Victoria/01/2020 stock

**Fig. 3.**
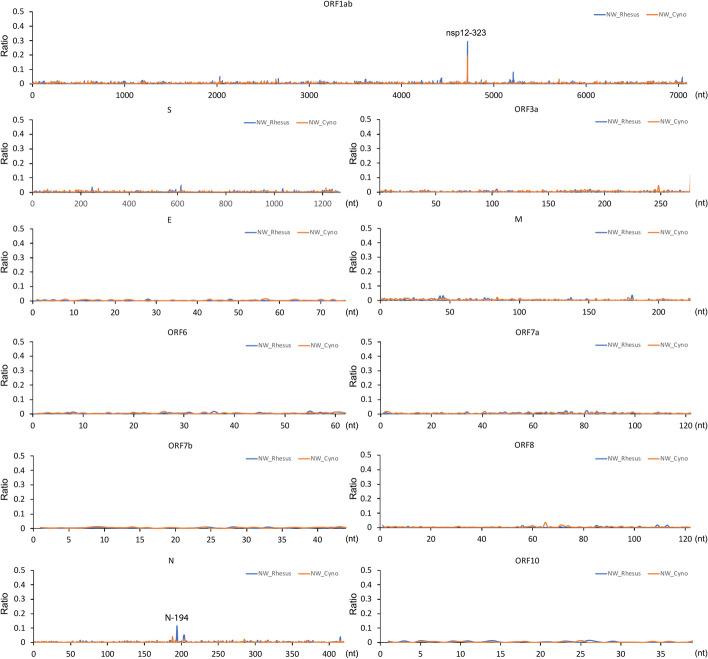
Analysis of minor variant genomes in cynomolgus (NW_Cyno; orange) and rhesus (NW_Rhesus; blue) macaques infected with the SARS-CoV-2 Victoria/01/2020 isolate using data from shotgun Illumina RNA sequencing of nasal washes (NW). Data is presented as a global average over the course of the infection from sequencing SARS-CoV-2 from longitudinal samples. Each SARS-CoV-2 open reading frame is indicated above the appropriate panel. The major difference was at position 323 in NSP12

Next, we looked at minor genomic variants in each animal from nasal washes obtained daily during infection [13]. RNA was purified and sequenced using two independent approaches, shotgun sequencing on an Illumina platform and via ARTIC-Illumina, with the latter for specifically sequencing SARS-CoV-2 RNA. To obtain a global overview and identify whether there were any hot spots for minor genomic variants, these were plotted as an average over the course of the infections in the NHPs (Fig. 3). The data indicated that minor genomic variants occurred throughout the genome, but the most frequent variation occurred at nucleotide position 14,408 in the ORF1AB region, which resulted in a C to U change. This resulted in a non-synonymous change in NSP12 with the substitution of P323L (amino acid position 4715 with respect to the ORF1AB polyprotein). The second most common variation was observed in the N gene at nucleotide position 28,854. This resulted in a non-synonymous amino acid change of S194L.

**Fig. 4.**
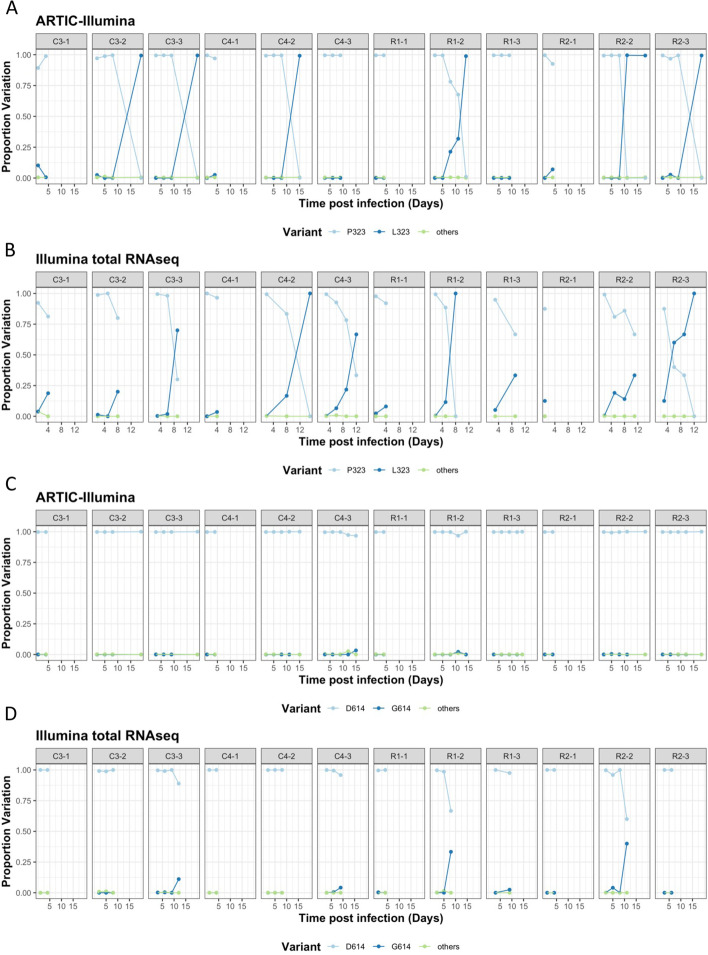
Analysis of NSP12 position 323 and spike position 614 through ARTIC-Illumina sequencing (A/C) and Illumina total RNAseq (B/D) from nasopharyngeal swabs taken longitudinally from infected cynomolgus (CX-X, *n*=6) and rhesus (RX-X, *n*=6) macaques. Data in this figure is from the ARTIC-Illumina approach to specifically amplify SARS-CoV-2 RNA (coverage filtered at 20×) and the Illumina total RNAseq approach without prior amplification (coverage filtered at 5×). The day post infection is shown for the animals. At position 323/614, a P/D is shown as light blue, an L/G as dark blue respectively, and green indicates other substitutions. The left-hand *y*-axis indicates the proportion of variation at the indicated position. (NHPs C= cynomolgus, R= rhesus macaque, CX-X/RX-X is the identity of the animal, with the experimental group C/RX and the animal number as -X)

To determine how rapidly the P323L and D614G substitutions were selected for in the individual animals, sequences from longitudinal samples were analyzed to investigate P323L and D614G (Fig. 4), showing both ARTIC-Illumina and total Illumina RNAseq approaches. The coverage was filtered at 20 in ARTIC-Illumina and 5 in total Illumina RNAseq, reflecting the differences in sequence read depth for each approach. The sequencing data, using the two different approaches, showed that the P323L mutation was already present as a minor genomic variant (at higher levels than the inoculum) by day 1 in some animals, as well as the presence of other minor genomic variants at this position. However, as infection progressed, the frequency of the L323 minor genomic variant increased and became part of the dominant viral genome sequence by the end point of infection. We note that this occurred in 6 out of 12 NHPs with samples sequenced using the ARTIC-Illumina approach and in 5 out of 12 NHPs with samples sequenced using the Illumina total RNAseq (Fig. 4). This may be due to the limited number of samples/sequencing information from these animals, reflecting a lack of later timepoints when such changes could be apparent. The spike minor genomic variant G614 was seen at low levels and did not become dominant over the wildtype D614 over the time course (Fig. 4C, D), in contrast to the P323L minor genomic variant.

### L323 has a strong selective advantage over P323 in the context of a clinical isolate with D614 dominant in the spike protein

**Fig. 5.**
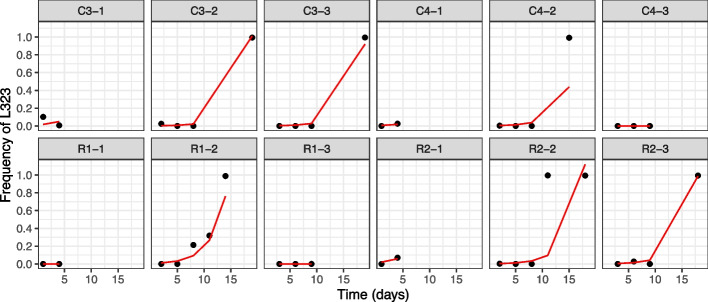
Predicted fits of the exponential growth model for the L323 substitution in 12 NHPs using the data shown in Fig. 4 for ARTIC-Illumina. The red line indicates the model fit estimated with a generalized linear mixed-effects model (GLMM), and black points correspond to frequency of L323 mutation over time. (NHPs C= cynomolgus, R= rhesus macaque, CX-X/RX-X is the identity of the animal)

To compare the relative fitness of each variant, the selection coefficient of P323 and L323 was derived from the NHP study of the abundance of genomes with P323 versus L323. This was analyzed using the ARTIC-Illumina sequencing data by a simple exponential growth model. The relative growth advantage of genomes with the L323 substitution was estimated to be 0.42 (95% confidence intervals 0.4185–0.4218) compared to the P323 variant (Fig. 5). This finding indicated that the L323 substitution conferred a strong selective advantage in a virus with the background of mostly D614 variant.

### The P323L substitution in NSP12 results in a change in plaque phenotype and increased viral RNA and protein synthesis in the context of viruses generated through reverse genetics with G614 in the spike protein

**Fig. 6.**
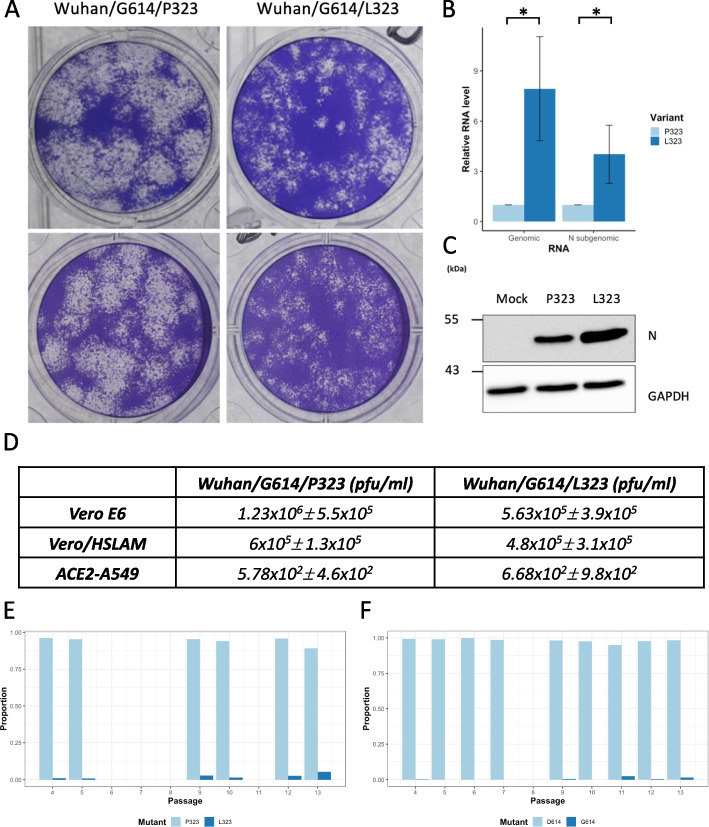
Investigating growth of P323 and L323 in cell culture. **A** Representative images of plaques formed by two viruses created through reverse genetics that have the Wuhan-Hu-1 background (NC_045512) and an engineered D614G substitution in the spike protein, and either P323 or L323 in NSP12 (termed Wuhan/G614/P323 and Wuhan/G614/L323 respectively). **B** Relative RNA levels of genomic or N subgenomic RNA with Wuhan/G614/P323 or Wuhan/G614/L323 from RT-qPCR on ACE2-A549 cells infected with either virus at 24h. Error bars show standard deviation. Unpaired *t*-tests without Welch’s correction, *p*=0.0181 and *p*=0.0393 respectively, for *n*=3 biological replicates. **C** Western blot analysis of the abundance of nucleoprotein produced in either mock infected, or cells infected with Wuhan/G614/P323 or Wuhan/G614/L323. This is an exemplar western blot for an experiment that was done in triplicate; GAPDH is shown as a protein loading control. **D** Mean viral titers (pfu/ml, *n*=3 biological replicates ± standard deviation) at 24hpi in Vero E6, Vero/hSLAM, and ACE2-A549 cells infected with either Wuhan/G614/P323 or Wuhan/G614/L323. **E,F** Proportion of amino acid P323/L323 in NSP12 (**E**) or D614/G614 in the Spike protein (**F**) in the Victoria/01/2020 isolate serially passaged through cells over 13 sequential passages (coverage filtered at 20×)

Previous data indicated that Victoria/01/2020 grew with a small plaque phenotype and lower titer compared to more contemporary variants including Variants of Concern (VOCs), which grew to higher titers with larger or mixed plaque morphologies [17]. The later virus isolates contained the P323L and D614G substitutions in NSP12 and the spike protein respectively, as the dominant viral genome sequence, as well as other changes. To investigate whether the L323 substitution conferred an advantage over and above the G614 change in the spike protein, two viruses were created through reverse genetics that were based on the Wuhan-Hu-1 virus (NC_045512) with a G614 background, one with a P323 (Wuhan/G614/P323) and the other with a L323 (Wuhan/G614/L323) in NSP12. The background context of a genome with G614 in spike was chosen because this substitution has been proposed to have occurred first or concurrently with P323L in NSP12 in variants assigned to lineage B [18]. Likewise, there is no evidence of persistence of D614 in the global population of SARS-CoV-2 as part of a dominant sequence. Growth of these two defined viruses was compared in Vero E6 cells by examining plaque morphology. The data indicated that Wuhan/G614/L323 had a smaller plaque phenotype (Fig. 6A), suggesting that the L323 substitution conferred altered viral properties. Therefore, to further investigate the potential differences between P323 and L323, the amount of intracellular viral RNA and protein was determined. ACE2-A549 cells were infected with either Wuhan/G614/P323 or Wuhan/G614/L323 at MOI of 0.5 for 24h. A one step growth curve indicated this time point was in the region of first viral release but not secondary or tertiary viral release [17]. Likewise, cell death could be observed at later time points and thus these would confound analysis. The abundance of the nucleocapsid gene (as determined by RT-PCR) and protein (as determined by western blot) for Wuhan/G614/L323 was greater than for Wuhan/G614/P323 virus (Fig. 6B, C). This suggested that increased viral replication/transcription was associated with L323 compared to P323. However, analysis of viral titers in Vero E6 cells at 24 h post infection (hpi) between the two defined viruses grown in either Vero E6, ACE2-A549 or Vero/hSLAM cells did not show a significant difference in titer between them—at least considering log fold changes (Fig. 6D).

To investigate whether there was a selective advantage of L323 over P323, the Victoria/01/2020 isolate was passaged ten times in ACE2-A549 cells, with sequencing used to investigate the proportion of P323 to L323 at each passage. The starting proportion of L323 was 0.0003% (Table 1) which after ten passages increased to 5.4% (Fig. 6E), suggesting a selective advantage of minor variant genomes with this substitution. The proportion of G614 also increased with passage (Fig. 6F).

### Maintenance of variation at position 323 in NSP12 in the population

**Fig. 7.**
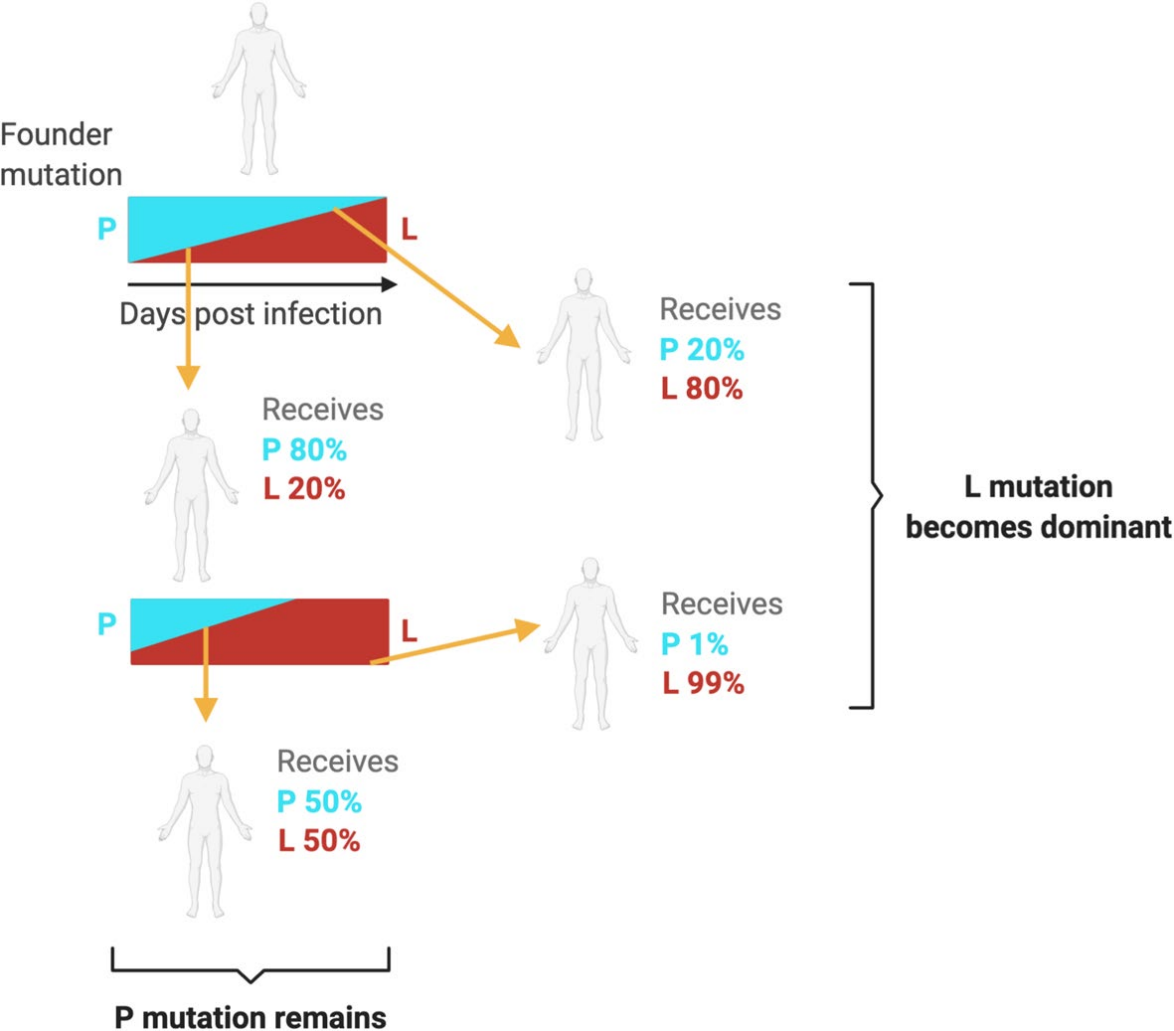
Model for the transmission of variant genomes. This model suggests that genomes encoding amino acids under strong selection pressure (such as P323 in this case) have potential options for growth and transmission of viral populations via either consensus viral genomes with P323 (cyan) and L323 (red) present in minor variant genomes or in equilibrium, or where L323 is dominant in the viral genome sequence and P323 present at a minor variant level. Given the potential strong selection pressure on position 323, the time post infection transmission occurs is crucial in determining which variant becomes dominant viral genome sequence. This figure was created using Biorender.com

Based on the experimental data presented in this study, we propose a model where the emergence and distribution of minor variant genomes and dominant viral genome sequence for SARS-CoV-2 is dependent on selection pressure and time post infection at which a virus population is transmitted onwards to another individual (Fig. 7).

**Fig. 8.**
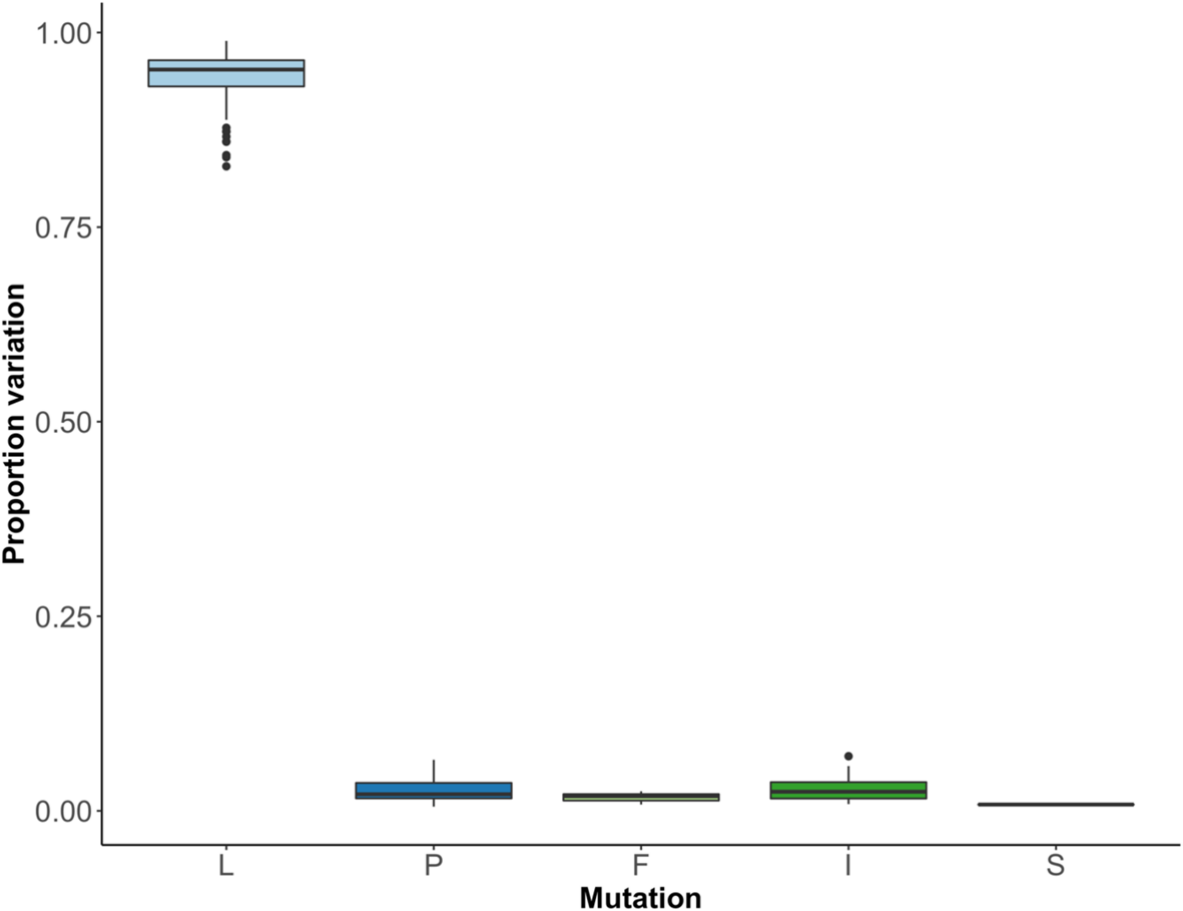
Amino acid mutations at site 323 in NSP12 in samples sequenced using the ARTIC-Nanopore approach (*n*=101) from July to September 2021 obtained from the Short Read Archive. The bioinformatics tool DiversiTools was used to generate proportions of the counts of amino acids at site 323 and showed that L is dominant in viral sequences from mid-late 2021, with P remaining a small proportion of the population alongside amino acids F, S, and I

One of the predictions of this model is that while L323 in NSP12 might now be part of the dominant viral genome sequence, other variants at this position will be present and persist (e.g., P323) at this position. To test this, random contemporary sequence data (post the P323L and D614G substitutions) that had been deposited between July and September 2021 on the Short Read Archive was examined for variation at position 323 in NSP12 (Fig. 8). The data indicated that L323 is the dominant variant, but P323 and other substitutions such as F323 are present as minor genomic variants.

## Discussion

Several variants have come to dominate the global landscape of SARS-CoV-2 infections, including ones with the initial D614G and P323L polymorphisms in the spike protein and NSP12 respectively (B.1), followed by Alpha (B.1.1.7), Delta (B.1.617.2), and Omicron (B.1.1529). These have occurred in waves and are likely linked to increases in transmissibility [4], coupled with spike variation-mediated immune escape [19, 20], founder effects [21–23], behavior patterns of hosts and population density [24, 25], and non-pharmaceutical interventions [26]. While VoCs have differed in terms of transmissibility, in general there has been no marked change in inherent morbidity and mortality, although an early variant with a deletion in ORF8 was associated with a less severe inflammatory response and better patient outcome [3]. Omicron BA.1 is potentially less severe, although this is hard to assess in the background of widespread prior immunity [27–29].

Among the first major changes in the dominant viral genome sequence of SARS-CoV-2 were the P323L and the D614G substitutions in NSP12 and the spike protein respectively. The apparent simultaneous appearances of these two mutations made it difficult to distinguish the biology of each mutation epidemiologically and focus has been placed on spike D614G and its association with increased infectivity and transmissibility [30, 31]. We wanted to investigate the selection pressure/contribution of P323L by analyzing the virus population in humans over the period when P323L and D614G became part of the dominant viral genome sequence, as well as studying this in two NHP animal models. The first analysis suggested rapid selection of P323L in NSP12 and D614G in the spike protein within humans. This was reflected in the substitutions L323 and G614 in the minor genomic variant population becoming the dominant viral genome sequence and replacing P323 and D614 within a few days of intra-host selection (Fig. 2). At the population level, data suggested that this selection was established over a 2-month period in the UK (February and March 2020). We note that although samples used in this study were collected early in the pandemic in the UK, during the containment phase and in the early surge phase of wave 1, there was no evidence that the P323L substitution in NSP12 and the D614G substitution in the spike protein resulted in an increase in disease severity or outcome [18, 30, 31].

The selection pressure at these two positions (within an isolate close to the original Wuhan outbreak) was evaluated in two NHP models for COVID-19 that recapitulate the acute infection with mild disease observed in most humans [13]. Here, the SARS-CoV-2 variant used for infection, Victoria/01/2020, had P323 in NSP12 and D614 in the spike protein in the dominant consensus sequence. At the minor variant genome level, L323 in NSP12 was present with a frequency of 0.03% and G614 in the spike protein at 0.02%. The sequence analysis indicated that for those animals where later time points returned usable viral genomic information, the dominant viral genome sequence now contained L323 in NSP12, but not necessarily G614 in the spike protein (Fig. 4). Minor genomic variation was also observed at amino acid position 194 in N, reflecting a population of amino acids present with S194 (~90%) and N194 (~10%) (Fig. 3). The N substitution S194L was detected as early as January 2020 in samples [32], arose widely in 2020 in many lineages but was not found to be present in VoCs including the most recent Omicron VoC lineages. S194L was found to be associated with symptomatic patients early in the pandemic in 2020 [33].

During the first stage of the COVID-19 pandemic, the global sequence landscape was dominated by lineage B; however, this was supplanted by lineage B.1 which shared both D614G and P323L. While there is evidence that D614G arose on multiple occasions, this was not the case with P323L and data suggested that P323L occurred concurrently with D614G [31]. The selection of P323L over D614G in the NHPs would suggest that in the viral isolate used, the L323 and G614 substitutions were present on separate minor variants, implying no linkage between the two mutations. It also suggests that there was little to no selection pressure to cause the emergence of D614G in this case, as even at the minor genomic variant level it remained in low abundance (Fig. 4). However, we note that our sequence characterization of the inoculum was by amplicon where we would not be able to identify if the two minor variants were present on the same genome. The differences in the accrual of these mutations may result from P323L being a host adapted mutation, whereas D614G emerged widely in the population, suggesting that it is a mutation associated with transmission between individuals.

No difference in growth (at least determined by plaque assay) was observed with the defined viruses containing P323 or L323, although in the plaque assays there was a visible difference in plaque morphology (Fig. 6A). Investigation of relative viral RNA and protein levels indicated that these products were more abundant in L323 compared to P323 viruses (Fig. 6B, C). While the D614G substitution plays a pivotal role in enhancing the growth of SARS-CoV-2, the contribution of P323L cannot be discounted.

Taken together with the analysis from the viruses created through reverse genetics, the exponential growth model applied to the NHP longitudinal study provides additional evidence that the P323L mutation is not neutral or mildly beneficial, but likely played a critical role in the rapid early emergence of the P323L/D614G genotype in the human population. The relative growth advantage is reflected through the selection coefficient of 0.42, suggesting strong selection of L323 over P323 and consequent maintenance in the viral population. This is also seen with passage of the Wuhan/G614/P323 wild type virus, which after 10 passages revealed increasing L over P at position 323. Therefore, rather than being a hitch-hiker substitution, P323L is likely to contribute to the selection of viruses with this phenotype.

NSP12 has been shown to attenuate type I interferon production [34], and this may be variant dependent. The mechanism behind the selection pressure acting on the P323L substitution in both humans and NHP animal models is unknown. However, NSP12 is the RNA-dependent RNA polymerase, and such polymerase complexes can be composed of both viral and host cell proteins [35, 36]. We speculate that the P323L substitution may alter the composition of the replication complex by altering interactions with the host cell proteome and thereby facilitating virus replication. Therefore, it is tempting to speculate that growth of viruses in cell lines from the original host species might revert the substitution back to wild type. This might provide a mechanism to narrow down candidates for the original zoonotic event(s).

During this study, our laboratories have used orthogonal approaches to investigate the potential differences in biology between genomes with P323 and genomes with L323. The data clearly illustrates that L323 possesses an advantage in vivo. However, our laboratory studies indicate that these advantages are hard to distinguish in vitro. In the context of SARS-CoV-2 lineages with the D614G substitution in the spike protein (and the P323L substitution in NSP12), we postulate that the dominant driver is the altered biology in spike, but nevertheless at a population level P323L also provides a small but significant selective advantage. However, interestingly in SARS-CoV-2 variants derived from lineage A, the D614G substitution occurred later than that in lineage B, and these viruses, such as A.19 and A.2.4 contained this substitution but not the P323L.

In our model (Fig. 7), an individual with the substitution present in a minor variant genome with a selective advantage will see an increase in the proportion of this genome as infection progresses. Under this selective pressure, the minor variant genome will become the dominant viral genome sequence. If transmission occurs early in infection, then the variant will be maintained at a minor genomic variant level. If selective pressure is strong, then the viral population that is being transmitted will have the substitution as part of the dominant viral genome sequence—and this will persist during further infections. Another consequence is that the sudden emergence of a substitution as part of the dominant genome sequence may be due to founder effect, for example, F323 in NSP12 that was identified in a cluster of cases in Northern Nevada and in Nigeria (B.1.525). However, this substitution has not become part of the global dominant viral genome sequence, despite F323 being identified in samples from early 2020.

L323 is now dominant in the viral population, as seen in Fig. 8 through sequencing analysis of ARTIC-Nanopore sequenced genomes deposited on the Short Read Archive in July–September 2021. While there is still a mixed population of amino acids at position 323, L is dominant with any other amino acid (including P), being at very low proportions less than 0.05. This reflects some conferred fitness advantage as the mutant P323L has been maintained across the viral population from May 2020 until at least September 2021 and is still present in global sequences as of June 2022, including VoCs and all recent Omicron lineages. The increase in transcription/replication compared to P323, of L323, seen here may be rooted in host-virus protein interactions.

## Conclusions

The data in this study indicates that, in some cases, it may be possible to predict the emergence of a new dominant viral genome sequence and hence new variant. This would be based on tracking the distribution and frequency of minor variant genomes at a population level, rather than just focusing on providing information on the dominant viral genome sequence, e.g., consensus-level reporting. While computationally more intensive and perhaps requiring higher-quality samples and sequencing data, the ability to earlier predict a newly emerging variant of SARS-CoV-2 with selective advantage in the global landscape may aid in the evaluation of medical countermeasures and non-pharmaceutical interventions.

## Materials and methods

### Illumina for NHP NW samples

Total RNA in each sample was extracted with QIAmp viral RNA extraction kit and eluted in pure water. Following the manufacturer’s protocols, total RNA was used as input material in to the QIAseq FastSelect –rRNA HMR (Qiagen) protocol to remove cytoplasmic and mitochondrial rRNA with a fragmentation time of 7 or 15 min. Subsequently, the NEBNext® Ultra™ II Directional RNA Library Prep Kit for Illumina® (New England Biolabs) was used to generate the RNA libraries, followed by 11 cycles of amplification and purification using AMPure XP beads. Each library was quantified using Qubit and the size distribution assessed using the Agilent 2100 Bioanalyser, and the final libraries were pooled in equimolar ratios. The raw FASTQ files (2 × 150 bp) generated by an Illumina® NovaSeq 6000 (Illumina®, San Diego, USA) were trimmed to remove Illumina adapter sequences using Cutadapt v1.2.1 [37]. The option “−O 3” was set, so the that 3′ end of any reads which matched the adapter sequence with greater than 3 bp was trimmed off. The reads were further trimmed to remove low-quality bases, using Sickle v1.200 [38] with a minimum window quality score of 20. After trimming, reads shorter than 10 bp were removed.

The minor variations of amino acid in the genes of virus were called as our previous description [39]. Hisat2 v2.1.0 [40] was used to map the trimmed reads on the cynomolgus (*M. fascicularis*) and rhesus (*M. mulatta*) reference genome assemblies (release-94) downloaded from the Ensembl FTP site. The unmapped reads were extracted by bam2fastq (v1.1.0) and then mapped on the inoculum SARS-CoV-2 genome (GenBank sequence accession: NC_045512.2) using Bowtie2 v2.3.5.1 [40] by setting the options to parameters “--local -X 2000 --no-mixed”, followed by SAM file to BAM file conversion, sorting, and removal of the reads with a mapping quality score below 11 using SAMtools v1.9 [41]. After that, the PCR and optical duplicate reads in the BAM files were discarded using the MarkDuplicates in the Picard toolkit v2.18.25 (http://broadinstitute.github.io/picard/) with the option of “REMOVE_DUPLICATES=true”. This BAM file was then processed by the diversiutils script in DiversiTools (http://josephhughes.github.io/btctools/) with the “-orfs” function to generate the number of amino acid changes caused by the nucleotide deviation at each site in the protein. In order to distinguish low-frequency variants from Illumina sequence errors, the diversiutils script used the calling algorithms based on the Illumina quality scores to calculate a *P*-value for each variant at each nucleotide site [42]. The amino acid change was then filtered based on the *P*-value (<0.05) to remove the low-frequency variants from Illumina sequence errors.

### ARTIC-Illumina for longitudinal swab samples and NHP NW samples

Samples from clinical specimens were processed at Containment Level 3 (CL3). Nasopharyngeal swabs were collected in viral transport media. Swabs were left to defrost in a Tripass I cabinet in CL3. The swab was removed from the tube and dipped in Virkon before disposal to reduce dripping and aerosol generation. Two hundred fifty milliliters of viral transport media was removed from the swab sample and added to 750ml of Trizol LS (Invitrogen (10296028)) and mixed well. Remaining extraction was continued under CL2 conditions. All RNA samples were then treated with Turbo DNase (Invitrogen). SuperScript IV (Invitrogen) was used to generate single-strand cDNA using random primer mix (NEB, Hitchin, UK). ARTIC V3 PCR amplicons from the single-strand cDNA were generated following the Nanopore Protocol of PCR tiling of SARS-CoV-2 virus (Version: PTC_9096_v109_revL_06Feb2020). The amplicon products were then used in Illumina NEBNext Ultra II DNA Library preparation. Following 4 cycles of amplification, the library was purified using Ampure XP beads and quantified using Qubit and the size distribution assessed using the Fragment Analyzer. Finally, the ARTIC library was sequenced on the Illumina® NovaSeq 6000 platform (Illumina®, San Diego, USA) following the standard workflow. The generated raw FASTQ files (2 × 250 bp) were trimmed to remove Illumina adapter sequences using Cutadapt v1.2.1 26. The option “−O 3” was set, so the that 3′ end of any reads which matched the adapter sequence with greater than 3 bp was trimmed off. The reads were further trimmed to remove low-quality bases, using Sickle v1.200 27 with a minimum window quality score of 20. After trimming, reads shorter than 10 bp were removed. The NHP NW total RNA have been extracted and sequenced in our previous paper [8].

The variation of amino acids in the genes of the virus were called as our previous description [39]. Hisat2 v2.1.0 [40] was used to map the trimmed reads onto the human reference genome assembly GRCh38 (release-91) downloaded from the Ensembl FTP site. The unmapped reads were extracted by bam2fastq (v1.1.0) and then mapped on a known SARS-CoV-2 genome (GenBank sequence accession: NC_045512.2) using Bowtie2 v2.3.5.1 [40] by setting the options to parameters “--local -X 500 --no-mixed”, followed by SAM file to BAM file conversion, sorting, and removal of the reads with a mapping quality score below 11, not in pair, and not primary and supplementary alignment using SAMtools v1.9 [41]. Bamclipper (v 1.0.0) [43] was used to trim the ARTIC primer sequences on the mapped reads within the BAM files. The reads without ARTIC primer sequences were also excluded in the further analysis. This trimmed BAM file was then processed by the diversiutils script in DiversiTools (http://josephhughes.github.io/DiversiTools/) with the “-orfs” function to generate the number of amino acid changes caused by the nucleotide deviation at each site in the protein in comparison to the reference SARS-CoV-2 genome (NC_045512.2). In order to distinguish low-frequency variants from Illumina sequence errors, the diversiutils script used the calling algorithms based on the Illumina quality scores to calculate a *P*-value for each variant at each nucleotide site [42].

### Rapid Sequencing Long Amplicons (RSLA) Nanopore for longitudinal swab samples

Total RNA of longitudinal swab samples was extracted as described above. Sequencing libraries for amplicons generated by RSLA [14] were prepared following the “PCR tiling of SARS-CoV-2 virus with Native Barcoding” protocol provided by Oxford Nanopore Technologies using LSK109 and EXP-NBD104/114. The artic-ncov2019 pipeline v1.2.1 (https://artic.network/ncov-2019/ncov2019-bioinformatics-sop.html) was used to filter the passed FASTQ files produced by Nanopore sequencing with lengths between 800 and 1600. This pipeline was then used to map the filtered reads on the reference SARS-CoV-2 genome (NC_045512.2) by minimap2 [44] and assigned each read alignment to a derived amplicon and excluded primer sequences based on the RSLA primer schemes in the BAM files. These BAM files were further analyzed using DiversiTools (http://josephhughes.github.io/DiversiTools/) with the “-orfs” function to generate the ratio of amino acid change in the reads and coverage at each site of the protein in comparison to the reference SARS-CoV-2 genome (NC_045512.2). The amino acids with highest ratio and coverage > 10 were used to assemble the consensus protein sequences.

### Sanger sequencing

cDNA template was amplified using Q5 High-Fidelity DNA Polymerase following the PCR conditions: denaturation at 98°C for 30 s followed by 39 cycles of 10 s denaturation at 98°C, 30 s annealing at 66°C, and then 50 s of extension at 72°C. A final extension step was done for 2 min at 72°C. The primer sets used for amplification were (SARS-CoV-2_15_LEFT=ATACGCCAACTTAGGTGAACG, SARS-CoV-2_15_RIGHT= AACATGTTG-TGCCAACCACC) to detect the P323L mutation or (SARS-CoV-2_24_LEFT= TTGAACTTCTACATGCACCAGC, SARS-CoV-2_RIGHT=CCAGAAGTGATTGTACCCGC) to detect the D614G mutation. PCR products were purified using AMPure XP beads (Beckman Coulter) and quantified using the Qubit High Sensitivity 1X dsDNA kit (Invitrogen). To visualize band quality, PCR products were run on a 1.5% agarose gel. Ten nanograms of each amplified product was sent for Sanger sequencing (Source Bioscience, UK).

### Cells

African green monkey kidney C1008 (Vero E6) cells (purchased from ATCC) were cultured in Dulbecco’s minimal essential medium (DMEM) (Sigma) with 10% fetal bovine serum (FBS) (Sigma) and 0.05mg/ml gentamicin at 37°C/5% CO_2_. Vero/hSLAM cells (UKHSA) were grown in DMEM with 10% FBS and 0.05mg/ml gentamicin (Merck) with the addition of 0.4mg/ml Geneticin (G418; Thermo Fisher) at 37°C/5% CO_2_. Vero E6 and Vero/hSLAM cells were authenticated at respective sources. Human ACE2-A549 (hACE2-A549), a lung epithelial cell line which overexpresses the ACE-2 receptor, were a kind gift of Oliver Schwartz [45] and cultured in DMEM with 10% FBS and 0.05mg/ml gentamicin with the addition of 10μg/ml Blasticidin (Invitrogen). Only passage 3–10 cultures were used for experiments. All cell lines were tested and were negative for mycoplasma contamination.

### Generation and culture of viruses with defined changes through reverse genetics

SARS-CoV-2 viruses with user defined changes were generated by reverse genetics using the “transformation-associated recombination” in yeast approach [46]. Eleven cDNA fragments with 70 bp end-terminal overlaps which spanned the entire SARS-CoV-2 isolate Wuhan-Hu-1 genome (GenBank accession: NC_045512) were produced by GeneArt™ synthesis (Invitrogen™, Thermo Fisher) as inserts in sequence verified, stable plasmid clones. The 5′ terminal cDNA fragment was modified to contain a T7 RNA polymerase promoter and an extra “G” nucleotide immediately upstream of the SARS-CoV-2 5′ sequence, while the 3′ terminal cDNA fragment was modified such that the 3′ end of the SARS-CoV-2 genome was followed by a stretch of 33 “A”s followed by the unique restriction enzyme site Asc I. The inserts were amplified by PCR using a Platinum SuperFi II mastermix (Thermo Fisher) and assembled into full-length SARS-CoV-2 cDNA clones in the YAC vector pYESL1 using a GeneArt™ High-Order Genetic Assembly System (A13285, Invitrogen™, Thermo Fisher) according to the manufacturer’s instructions. RNA transcripts produced from the YAC clones by transcription with T7 polymerase were used to recover infectious virus. Two viruses were produced on the Wuhan-Hu-1 background and had a D614G substitution in the spike protein and differed at amino acid position 323 in NSP12 with either a P or L, these were termed Wuhan/G614/P323 and Wuhan/G614/L323, respectively. Whole genome sequencing confirmed the presence of these changes. Stocks of the viruses were cultured in Vero E6 cells in DMEM containing 2% FBS and 0.05mg/ml gentamicin and harvested 72 h post inoculation. Virus stocks were aliquoted and stored at −80°C. All stocks were titred by plaque assay on Vero E6 cells and pictures of the resulting plaques recorded.

### Quantitative real-time PCR

RNA samples from ACE2-A549 infected cells were extracted using TRIzol, and gDNA was removed using Turbo DNase (Invitrogen). Two hundred nanograms from each sample was converted to cDNA using LunaScript RT SuperMix kit (NEB) and diluted 1:4 with DNase/RNase-free water. Gene expressions were measured using iTaq Universal SYBR Green Supermix (Bio-Rad). Viral genome or subgenomic mRNA levels were normalized to β-Actin using 2^−ΔΔCT^. Primer sequences and locations were used as previously described [47].

### SDS polyacrylamide gel electrophoresis (SDS-PAGE)

ACE2-A549 infected cells were lysed with RIPA lysis buffer supplemented with a protease inhibitor cocktail. Ten micrograms of cell lysate was boiled in 4× sample buffer at 70°C for 10 min and separated by 10% SDS-PAGE for 1h at 150V. Proteins were transferred onto polyvinylidene difluoride (PVDF) membranes. The blots were blocked in 5% non-fat milk in 0.1% 1× TBST. Primary antibodies were diluted in 5% non-fat milk in 0.1% 1× TBST and incubated overnight at 4°C. Primary antibodies were removed, and blots were washed for 5 min with 0.1% 1× TBST 3 times each. Then, horseradish peroxidase-conjugated secondary antibodies were diluted and incubated at room temperature for 1h followed by three washes. Blots were developed using enhanced chemiluminesence (ECL) and imaged using a ChemiDoc touch gel imaging system (Bio-Rad).

### Fitting an exponential growth model

For each subject, we extracted the mutational counts at site 323 per time point if the coverage at the site was at least 100 reads. To estimate the selection coefficient of the L323 mutation relative to the wild type state, P323, we fitted an exponential growth curve to L323 count data over time with a generalized linear mixed-effects model (GLMM) with log-link function using lme4 library [48] in R v 4.1.1 (https://www.R-project.org/). This approach allows for inter-subject variability by modelling differences between subjects as a random effect. Additionally, we excluded count data where coverage at the site was below 100. The 95% confidence intervals were estimated using a bootstrapping approach with the “confint” function from R “stats” library (https://www.R-project.org/). The predicted fits to the data were visualized using the “tidyverse” package [49].

### Serial passage of SARS-CoV-2 Victoria/01/2020

SARS-CoV-2 Victoria/01/2020 was passaged three times in Vero/hSLAM cells prior to receiving it. hACE2-A549 cells were then infected at an MOI of 0.01 and incubated for 72 h (Passage 4). Following this, 100μl was passaged to fresh cells and incubated at 37°C for 1 h. After the incubation, media was topped up with DMEM containing 2% FBS and 0.05mg/ml gentamicin and incubated for 72 h (Passage 5). This process was repeated until Passage 13 (a total of ten passages through hACE2-A549 cells).

### Analysis of a subset of global sequences from July to September 2021

Sequences were obtained from the Short Read Archive (SRA) under accession numbers: ERR6343731, ERR6343734, ERR6343745, ERR6343747, ERR6343749, ERR6344225, ERR6346453, ERR6346456, ERR6346459, ERR6758978, ERR6758981, ERR6759296, ERR6761288, ERR6761458, ERR6761562, ERR6761570, ERR6761711, ERR6761986, ERR6762387, ERR6762545, ERR6762546, ERR6825821, ERR6878898, ERR6879599, ERR6879604, ERR6887797, ERR6887811, ERR6887812, ERR6887820, ERR6888048, ERR6888063, ERR6888078, ERR6888265, ERR6888283, SRR16376487, SRR16376490, SRR16376491, SRR16376494, SRR16376495, SRR16376496, SRR16376497, SRR16376501, SRR16376502, SRR16376505, SRR16376510, SRR16376515, SRR16376516, SRR16376522, SRR16376523, SRR16376524, SRR16376526, SRR16376529, SRR16376530, SRR16376531, SRR16376536, SRR16376540, SRR16376543, SRR16376544, SRR16376547, SRR16376551, SRR16376552, SRR16376554, SRR16376557, SRR16376559, SRR16376573, SRR16376580, SRR16376589, SRR16376599, SRR16376608, SRR16376613, SRR16376614, SRR16376648, SRR16376678, SRR16376782, SRR16376802, SRR16376804, SRR16376807, SRR16376810, SRR16376884, SRR16376904, SRR16376907, SRR16376912, SRR16376913, SRR16376914, SRR16376916, SRR16376921, SRR16376922, SRR16376925, SRR16376927, SRR16376928, SRR16376929, SRR16376932, SRR16376935, SRR16376939, SRR16376940, SRR16376941, SRR16376943, SRR16376944, SRR16376946, SRR16376949, SRR16376951. All sequences were ARTIC-Nanopore sequenced using the V3 primer scheme and downloaded as SRA files. The SRA files were converted to FASTQ files using the SRA Toolkit v2.11.3 (https://github.com/ncbi/sra-tools) command fastq-dump. The FASTQ files were processed through the artic-ncov2019 v1.2.1 pipeline (https://artic.network/ncov-2019/ncov2019-bioinformatics-sop.html) and analyzed with DiversiTools as above.

## Supplementary Information

**Additional file 1.** Accession data used in the analysis presented in Fig. 2. This additional information consists of sequencing data of SARS-CoV-2 from the UK and worldwide.

**Additional file 2.** Uncropped images for the blots in Fig. 6.

**Additional file 3.** Review history.

## Data Availability

One of the datasets supporting the conclusions of this article is available in the National Center for Biotechnology Information repository, under the project accession number PRJNA789459 and can be accessed via https://www.ncbi.nlm.nih.gov/bioproject/PRJNA789459 [50]. The other datasets supporting the conclusions of this article are included within the article as Additional File 1.

## References

[1] Worobey M, Pekar J, Larsen BB, Nelson MI, Hill V, Joy JB, Rambaut A, Suchard MA, Wertheim JO, Lemey P (2020). The emergence of SARS-CoV-2 in Europe and North America. Science.

[2] Davidson AD, Williamson MK, Lewis S, Shoemark D, Carroll MW, Heesom KJ, Zambon M, Ellis J, Lewis PA, Hiscox JA, Matthews DA (2020). Characterisation of the transcriptome and proteome of SARS-CoV-2 reveals a cell passage induced in-frame deletion of the furin-like cleavage site from the spike glycoprotein. Genome Med.

[3] Young BE, Fong SW, Chan YH, Mak TM, Ang LW, Anderson DE, Lee CY, Amrun SN, Lee B, Goh YS (2020). Effects of a major deletion in the SARS-CoV-2 genome on the severity of infection and the inflammatory response: an observational cohort study. Lancet.

[4] Hou YJ, Chiba S, Halfmann P, Ehre C, Kuroda M, Dinnon KH (2020). SARS-CoV-2 D614G variant exhibits efficient replication ex vivo and transmission in vivo. Science.

[5] Yang HC, Chen CH, Wang JH, Liao HC, Yang CT, Chen CW (2020). Analysis of genomic distributions of SARS-CoV-2 reveals a dominant strain type with strong allelic associations. Proc Natl Acad Sci U S A.

[6] Simmonds P. Rampant C-->U hypermutation in the genomes of SARS-CoV-2 and other coronaviruses: causes and consequences for their short- and long-term evolutionary trajectories. mSphere. 2020;5(3):e00408–00420. 10.1128/mSphere.00408-20 PMC7316492 32581081

[7] Ratcliff J, Simmonds P (2021). Potential APOBEC-mediated RNA editing of the genomes of SARS-CoV-2 and other coronaviruses and its impact on their longer term evolution. Virology.

[8] Dong X, Penrice-Randal R, Goldswain H, Prince T, Randle N, Donovan-Banfield I (2022). Analysis of SARS-CoV-2 known and novel subgenomic mRNAs in cell culture, animal model, and clinical samples using LeTRS, a bioinformatic tool to identify unique sequence identifiers. Gigascience.

[9] Peacock TP, Penrice-Randal R, Hiscox JA, Barclay WS. SARS-CoV-2 one year on: evidence for ongoing viral adaptation. J Gen Virol. 2021;102(4):001584. 10.1099/jgv.0.001584 PMC8290271 33855951

[10] Lythgoe KA, Hall M, Ferretti L, de Cesare M, MacIntyre-Cockett G, Trebes A, et al. SARS-CoV-2 within-host diversity and transmission. Science. 2021;372(6539):eabg0821. 10.1126/science.abg0821 PMC8128293 33688063

[11] Dowall SD, Matthews DA, Garcia-Dorival I, Taylor I, Kenny J, Hertz-Fowler C, Hall N, Corbin-Lickfett K, Empig C, Schlunegger K (2014). Elucidating variations in the nucleotide sequence of Ebola virus associated with increasing pathogenicity. Genome Biol.

[12] Dong X, Munoz-Basagoiti J, Rickett NY, Pollakis G, Paxton WA, Günther S, Kerber R, Ng LFP, Elmore MJ, Magassouba N (2020). Variation around the dominant viral genome sequence contributes to viral load and outcome in patients with Ebola virus disease. Genome Biol.

[13] Salguero FJ, White AD, Slack GS, Fotheringham SA, Bewley KR, Gooch KE, Longet S, Humphries HE, Watson RJ, Hunter L (2021). Comparison of rhesus and cynomolgus macaques as an infection model for COVID-19. Nat Commun.

[14] Moore SC, Penrice-Randal R, Alruwaili M, Randle N, Armstrong S, Hartley C (2020). Amplicon-based detection and sequencing of SARS-CoV-2 in nasopharyngeal swabs from patients with COVID-19 and identification of deletions in the viral genome that encode proteins involved in interferon antagonism. Viruses.

[15] Nasir JA, Kozak RA, Aftanas P, Raphenya AR, Smith KM, Maguire F (2020). A comparison of whole genome sequencing of SARS-CoV-2 using amplicon-based sequencing, random hexamers, and bait capture. Viruses.

[16] Caly L, Druce J, Roberts J, Bond K, Tran T, Kostecki R, Yoga Y, Naughton W, Taiaroa G, Seemann T (2020). Isolation and rapid sharing of the 2019 novel coronavirus (SARS-CoV-2) from the first patient diagnosed with COVID-19 in Australia. Med J Aust.

[17] Prince T, Dong X, Penrice-Randal R, Randle N, Hartley C, Goldswain H, et al. Analysis of SARS-CoV-2 in nasopharyngeal samples from patients with COVID-19 illustrates population variation and diverse phenotypes, placing the growth properties of variants of concern in context with other lineages. mSphere. 2022;7(3):e00913–00921. 10.1128/msphere.00913-21 PMC9241508 35491827

[18] Ilmjarv S, Abdul F, Acosta-Gutierrez S, Estarellas C, Galdadas I, Casimir M, Alessandrini M, Gervasio FL, Krause KH (2021). Concurrent mutations in RNA-dependent RNA polymerase and spike protein emerged as the epidemiologically most successful SARS-CoV-2 variant. Sci Rep.

[19] Wang B, Goh YS, Prince T, Ngoh EZX, Salleh SNM, Hor PX, Loh CY, Fong SW, Hartley C, Tan SY (2021). Resistance of SARS-CoV-2 variants to neutralization by convalescent plasma from early COVID-19 outbreak in Singapore. NPJ Vaccines.

[20] Saad-Roy CM, Morris SE, Metcalf CJE, Mina MJ, Baker RE, Farrar J, Holmes EC, Pybus OG, Graham AL, Levin SA (2021). Epidemiological and evolutionary considerations of SARS-CoV-2 vaccine dosing regimes. Science.

[21] Gomez-Carballa A, Bello X, Pardo-Seco J, Martinon-Torres F, Salas A (2020). Mapping genome variation of SARS-CoV-2 worldwide highlights the impact of COVID-19 super-spreaders. Genome Res.

[22] Diez-Fuertes F, Iglesias-Caballero M, Garcia-Perez J, Monzon S, Jimenez P, Varona S, et al. A founder effect led early SARS-CoV-2 transmission in Spain. J Virol. 2021;95(3):e01583–20. 10.1128/JVI.01583-20 PMC7925114 33127745

[23] Tasakis RN, Samaras G, Jamison A, Lee M, Paulus A, Whitehouse G, Verkoczy L, Papavasiliou FN, Diaz M (2021). SARS-CoV-2 variant evolution in the United States: High accumulation of viral mutations over time likely through serial Founder Events and mutational bursts. PLoS One.

[24] Ward T, Glaser A, Johnsen A, Xu F, Hall I, Pellis L (2021). Growth, reproduction numbers and factors affecting the spread of SARS-CoV-2 novel variants of concern in the UK from October 2020 to July 2021: a modelling analysis. BMJ Open.

[25] Rader B, Scarpino SV, Nande A, Hill AL, Adlam B, Reiner RC, Pigott DM, Gutierrez B, Zarebski AE, Shrestha M (2020). Crowding and the shape of COVID-19 epidemics. Nat Med.

[26] Kraemer MUG, Hill V, Ruis C, Dellicour S, Bajaj S, McCrone JT, Baele G, Parag KV, Battle AL, Gutierrez B (2021). Spatiotemporal invasion dynamics of SARS-CoV-2 lineage B.1.1.7 emergence. Science.

[27] Veneti L, Boas H, Brathen Kristoffersen A, Stalcrantz J, Bragstad K, Hungnes O, et al. Reduced risk of hospitalisation among reported COVID-19 cases infected with the SARS-CoV-2 Omicron BA.1 variant compared with the Delta variant, Norway, 2021 to January 2022. Euro Surveill. 2022;27(4):pii=2200077. 10.2807/1560-7917.ES.2022.27.4.2200077 PMC8796289 35086614

[28] Abdullah F, Myers J, Basu D, Tintinger G, Ueckermann V, Mathebula M, Ramlall R, Spoor S, de Villiers T, Van der Walt Z (2022). Decreased severity of disease during the first global omicron variant covid-19 outbreak in a large hospital in tshwane, south africa. Int J Infect Dis.

[29] Wolter N, Jassat W, Walaza S, Welch R, Moultrie H, Groome M, Amoako DG, Everatt J, Bhiman JN, Scheepers C (2022). Early assessment of the clinical severity of the SARS-CoV-2 omicron variant in South Africa: a data linkage study. Lancet.

[30] Korber B, Fischer WM, Gnanakaran S, Yoon H, Theiler J, Abfalterer W, Hengartner N, Giorgi EE, Bhattacharya T, Foley B (2020). Tracking changes in SARS-CoV-2 spike: evidence that D614G increases infectivity of the COVID-19 virus. Cell.

[31] Volz E, Hill V, McCrone JT, Price A, Jorgensen D, O'Toole Á (2021). Evaluating the effects of SARS-CoV-2 spike mutation D614G on transmissibility and pathogenicity. Cell.

[32] Khailany RA, Safdar M, Ozaslan M (2020). Genomic characterization of a novel SARS-CoV-2. Gene Rep.

[33] Barona-Gomez F, Delaye L, Diaz-Valenzuela E, Plisson F, Cruz-Perez A, Diaz-Sanchez M, et al. Phylogenomics and population genomics of SARS-CoV-2 in Mexico during the pre-vaccination stage reveals variants of interest B.1.1.28.4 and B.1.1.222 or B.1.1.519 and the nucleocapsid mutation S194L associated with symptoms. Microb Genom. 2021;7:000684. 10.1099/mgen.0.000684 PMC8743546 34846283

[34] Wang W, Zhou Z, Xiao X, Tian Z, Dong X, Wang C, Li L, Ren L, Lei X, Xiang Z, Wang J (2021). SARS-CoV-2 nsp12 attenuates type I interferon production by inhibiting IRF3 nuclear translocation. Cell Mol Immunol.

[35] Munday DC, Wu W, Smith N, Fix J, Noton SL, Galloux M, Touzelet O, Armstrong SD, Dawson JM, Aljabr W (2015). Interactome analysis of the human respiratory syncytial virus RNA polymerase complex identifies protein chaperones as important cofactors that promote L-protein stability and RNA synthesis. J Virol.

[36] Noton SL, Aljabr W, Hiscox JA, Matthews DA, Fearns R (2014). Factors affecting de novo RNA synthesis and back-priming by the respiratory syncytial virus polymerase. Virology.

[37] Martin M (2011). Cutadapt removes adapter sequences from high-throughput sequencing reads. EMBnet J.

[38] Joshi N, Fass J (2011). Sickle: A sliding-window, adaptive, quality-based trimming tool for FastQ files (Version 1.33)[Software].

[39] Dong X, Munoz-Basagoiti J, Rickett NY, Pollakis G, Paxton WA, Günther S, Kerber R, Ng LF, Elmore MJ (2020). Magassouba Nf: Variation around the dominant viral genome sequence contributes to viral load and outcome in patients with Ebola virus disease. Genome Biol.

[40] Kim D, Langmead B, Salzberg SL (2015). HISAT: a fast spliced aligner with low memory requirements. Nat Methods.

[41] Li H, Handsaker B, Wysoker A, Fennell T, Ruan J, Homer N, Marth G, Abecasis G, Durbin R (2009). The sequence alignment/map format and SAMtools. Bioinformatics.

[42] Morelli MJ, Wright CF, Knowles NJ, Juleff N, Paton DJ, King DP, Haydon DT (2013). Evolution of foot-and-mouth disease virus intra-sample sequence diversity during serial transmission in bovine hosts. Vet Res.

[43] Au CH, Ho DN, Kwong A, Chan TL, Ma ES (2017). BAMClipper: removing primers from alignments to minimize false-negative mutations in amplicon next-generation sequencing. Sci Rep.

[44] Li H (2018). Minimap2: pairwise alignment for nucleotide sequences. Bioinformatics.

[45] Buchrieser J, Dufloo J, Hubert M, Monel B, Planas D, Rajah MM, Planchais C, Porrot F, Guivel-Benhassine F, Van der Werf S (2020). Syncytia formation by SARS-CoV-2-infected cells. EMBO J.

[46] Thi Nhu Thao T, Labroussaa F, Ebert N, V'Kovski P, Stalder H, Portmann J, Kelly J, Steiner S, Holwerda M, Kratzel A (2020). Rapid reconstruction of SARS-CoV-2 using a synthetic genomics platform. Nature.

[47] White KM, Rosales R, Yildiz S, Kehrer T, Miorin L, Moreno E, Jangra S, Uccellini MB, Rathnasinghe R, Coughlan L (2021). Plitidepsin has potent preclinical efficacy against SARS-CoV-2 by targeting the host protein eEF1A. Science.

[48] Bates D, Mächler M, Bolker B, Walker S (2015). Fitting linear mixed-effects models using lme4. J Stat Software.

[49] Wickham H, Averick M, Bryan J, Chang W, D'Agostino McGowan L, François R, Grolemund G, Hayes A, Henry L, Hester J (2022). Welcome to the Tidyverse. J Open Source Software.

[50] Goldswain H, Dong X, Penrice-Randal R, Alruwaili M, Shawli GT, Prince T, et al. The P323L substitution in the SARS-CoV-2 polymerase (NSP12) has a selective advantage in humans and non-human primates that correlates with a change in phenotype in vitro. Sequence Read Arch https://www.ncbi.nlm.nih.gov/bioproject/PRJNA789459/. 2022.

