# Supplementary figures and images for: The P323L substitution in the SARS-CoV-2 polymerase (NSP12) confers a selective advantage during infection
Source: Genome Biol. 2023 Mar 13;24:47. doi: 10.1186/s13059-023-02881-5 (PMC10009825; doi:10.1186/s13059-023-02881-5)

Mock P323 L323

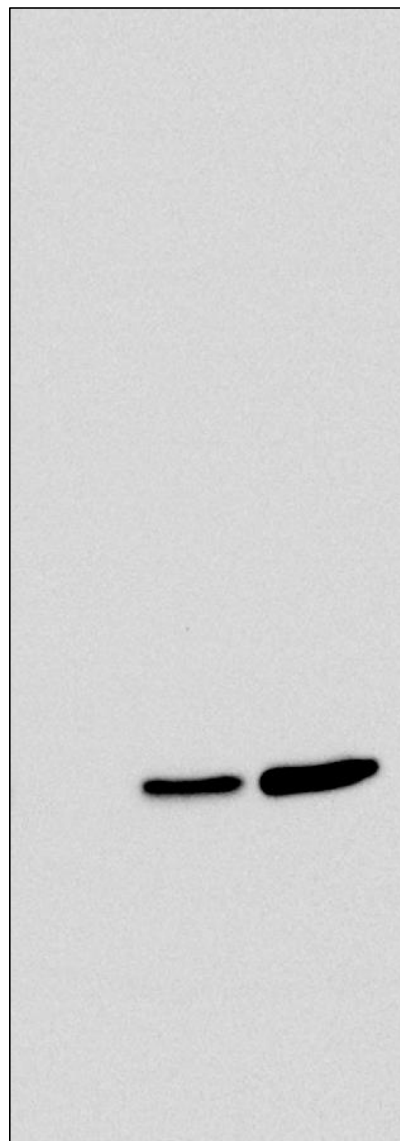

N

Mock P323 L323

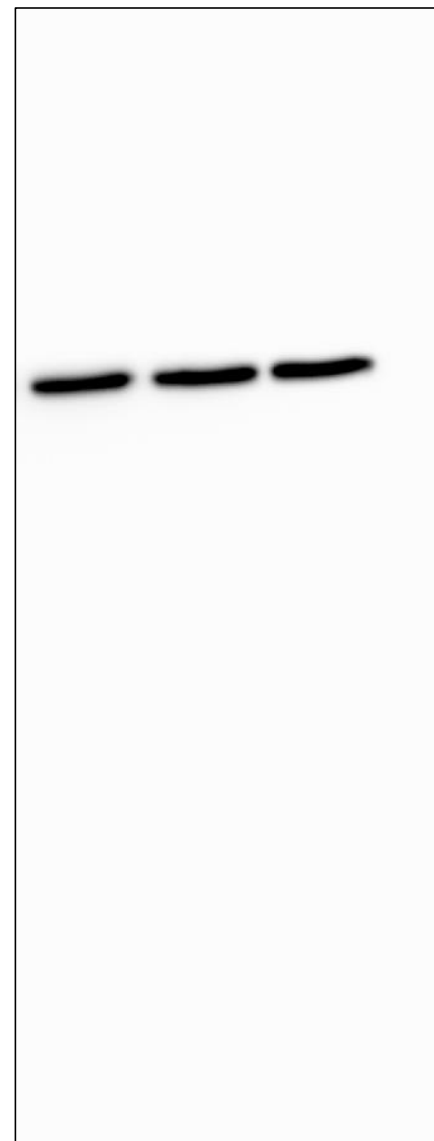

GAPDH

Supplement: Supplementary file 2 — Additional file 2. Uncropped images for the blots in Fig. 6. [file 13059_2023_2881_MOESM2_ESM.pdf]
