# Supplementary material for: The P323L substitution in the SARS-CoV-2 polymerase (NSP12) confers a selective advantage during infection
Source: Genome Biol. 2023 Mar 13;24:47. doi: 10.1186/s13059-023-02881-5 (PMC10009825; doi:10.1186/s13059-023-02881-5)
Supplement: Supplementary file 3 — Additional file 3. Review history. [file 13059_2023_2881_MOESM3_ESM.docx]

Review History

**First round of review**

**Reviewer 1**

**Were you able to assess all statistics in the manuscript, including the appropriateness of statistical tests used?**

Yes, and I have assessed the statistics in my report.

**Comments to author:**

In their manuscript, Goldswain et al. investigate the minor frequency viral mutations in SARS2 at a population level, within non-human primates and in vitro cell cultures. A change from P to L at site 323 in the viral polymerase NSP12 at the population level accompanied a change in the spike protein at site 614 from D to G and they show that this change was predictable based on the minor variants seen within the early samples in February-March 2020. The D614G spike change is known to increase transmissibility, but the authors also show that 323L contributed to the transmission advantage and that the amino acid change has a clear effect on the virus phenotype in vitro.

The methods used are appropriate and sufficiently detailed to allow replication; the results are supported by the data shown. The paper is well written and will be of broad interest. However, considering the broad readership of Genome Biology, I think that the use of the term "recombinant" is inappropriate in this context. I know that this is the way that lab constructs with different mutations are often referred to, but this will cause a lot of confusion as the term refers to a virus genome that has undergone the evolutionary process of recombination. The word should either be changed or very clearly explained by the authors the first time it is used.

Additionally:

Figure 2: annotate the figure for P323, 323L, D614 and 614G.

Figure 4: annotate the amino acid position on the figure (maybe on the figure legend)

Line 347: "there was no evidence that the change from P323L in NSP12 and D614G in the spike protein resulted in an increase in disease severity" => add a citation

The bioproject data is not yet public.

**Reviewer 2**

**Were you able to assess all statistics in the manuscript, including the appropriateness of statistical tests used?**

No, I do not feel adequately qualified to assess the statistics.

**Comments to author:**

Goldswain et al investigated the apparent co-occurrence of P323L in NSP12 and D614G in spike and argued that P323L may play a role in viral transmission. They went on to show perhaps unexpectedly only P323L was preferentially selected in vivo but not D614G despite both being present with a similar proportion in the virus stock used. They then chose to characterise P323 and 323L in vitro on a D614G backbone and concluded that 323L may contribute to enhanced virus transmission. Overall, the genomic analyses seem to be robust with multiple sequencing methods applied and good threshold cut-offs chosen. However, the analysis could be much strengthened by including early collected samples worldwide, especially before wave 1 where only 9 samples were at present analysed. Proper controls are required to support their claims for in vitro studies.

Major points:

It would be useful to provide the frequencies of minor populations in spike and NSP12 from the intra-patient samples before and after the appearance of the double mutants. This could provide some clues for the fast evolution of P323L and D614G (within two days). Depending on its frequency, it may also provide some evidence on why the double mutants, being a minor population, failed to evolve in vivo simultaneously in animal studies. This is important since the authors would like to make a case for monitoring the minor viral population in predicting the outcome of future variants.

The authors wanted to make a case that the double mutants co-occurred despite the animal studies suggesting otherwise. Then they went on to make recombinant viruses based on D614G. The reason for choosing 614G over D614 needs to be justified.

Line 225-231: The authors argued that there was an increase in L across all the animals even post 1 day inoculation and this population became dominant by the end of infection in all animals. However, Figure 4 suggests this is not the case in particular in animals C4 and R4 whereby the dominant was still P on day 5.

Line 273: The virus titer needs to be shown; also to provide the type of cells on which the plaque assays were conducted. Figure 6 suggests that despite 323L replicating faster the infectious titer is unaltered post 24 hr of infection. It is plausible that this phenotype is due to the non-productive replication of 323L bearing NSP12 and/or the forming of non-infectious virions in the presence of 323L. This needs to be extended in the discussion. But the experiment addressing the question of whether or not 323L confers a selective advantage would be to infect the cells with mixed P323 and 323L viruses followed by quantitating the virus population over time.

Line 280: It was assumed 614G conferred more advantage over D614 in viral transmission but this difference was not directly compared in their system. Hence it is incorrect to say the difference in viral RNA/protein is 'marginal' in comparison to the difference seen in the acquisition of D614G.

Minor points:

The codes for animals should be consistent in Figures 4 and 5. The labels on the X axes should be consistent for Figure 4.

Explain why there are more time points in the same collection for two sequence analyses. For example, animal C3 had 3 collection points for ARTIC but 4 for total RNAseq.

It is clear that the appearance of 323L is not dependent on the occurrence of D614G in those animals. Is there any evidence to show the appearance of 323L preceded 614G in humans?

Figure 5: There are two types of frequencies as shown in Figure 4. Clarification of which frequencies were used in this analysis and the reasons why they were chosen is needed.

Figure 6B: It would be useful to show the location of the primer pairs for genomic and subgenomic on the viral genome. Do the error bars represent the technical replicates from one experiment or multiple experiments?

**Reviewer 3**

**Were you able to assess all statistics in the manuscript, including the appropriateness of statistical tests used?**

No, I do not feel adequately qualified to assess the statistics.

**Comments to author:**

This interesting study by Goldswain et.al. convincingly show that the P323L substitution in the SARS-CoV-2 RNA-dependent RNA polymerase (NSP12) gradually dominates the virus population. The strength of the study is the use of powerful sequencing analyses in addition to sound epidemiology. The authors also performed experiments with the hope to bolster the claim that P323L substitution confers growth advantage to the virus. However, this part of the study may be improved by performing a couple of straightforward experiments. My specific comments are as following:

Lines 69-70: The authors wrote " During infection there was rapid selection of 323L into the dominant viral genome sequence but not 614G. In cell culture, recombinant viruses with either P323 or 323L in NSP12 had different phenotypes"

It would be more straightforward if the authors directly describe the actual differences rather than saying "had difference phenotypes".

Lines 120-122: same as above comment.

Figure 3: Please describe what the minor variants observed in the N gene are.

Lines 192-194: Again, please explain how the experiment was performed first. MOI, time???

Line 244-245: The authors stated "323L has a strong selective advantage over P323 in the context of a recombinant virus with the 245 D614 spike variant".

This paragraph needs some expansion to explain how the analysis was performed. There was no description of the recombinant virus. Please provide some experimental details here.

Lines 226-228: would it be possible for the authors to run PCR and subclone the fragments for Sanger sequencing to prove P323L mutation was already present as a minor genomic variant by Day 1 in some animals?

Figure 6. Please consider performing additional experiments for panel B and C by including more than one time point if the authors intend to detect any differences in growth kinetics.

**Authors Response**

**Point-by-point responses to the reviewers’ comments:**

Reviewer #1:

In their manuscript, Goldswain et al. investigate the minor frequency viral mutations in SARS2 at a population level, within non-human primates and in vitro cell cultures. A change from P to L at site 323 in the viral polymerase NSP12 at the population level accompanied a change in the spike protein at site 614 from D to G and they show that this change was predictable based on the minor variants seen within the early samples in February-March 2020. The D614G spike change is known to increase transmissibility, but the authors also show that 323L contributed to the transmission advantage and that the amino acid change has a clear effect on the virus phenotype in vitro.

The methods used are appropriate and sufficiently detailed to allow replication; the results are supported by the data shown. The paper is well written and will be of broad interest. However, considering the broad readership of Genome Biology, I think that the use of the term "recombinant" is inappropriate in this context. I know that this is the way that lab constructs with different mutations are often referred to, but this will cause a lot of confusion as the term refers to a virus genome that has undergone the evolutionary process of recombination. The word should either be changed or very clearly explained by the authors the first time it is used.

*Reply: We agree with the reviewer for the clarification of the term ‘recombinant’. We have changed this term to viruses created through/by reverse genetics – or a similar phrase(s).*

*Although not in response to this reviewer or the other reviewers we have changed our notation for P323/323L as single amino acids - we put the amino acid in front the numeral position, to standardise this with other literature.*

Additionally:

Figure 2: annotate the figure for P323, 323L, D614 and 614G.

*Reply: We considered annotating the figure but instead have added further explanatory text in the legend for Figure 2 which we hope clarifies this better.*

Figure 4: annotate the amino acid position on the figure (maybe on the figure legend)

*Reply: We have changed the annotation of the amino acid position on the Figure as suggested by the reviewer.*

Line 347: "there was no evidence that the change from P323L in NSP12 and D614G in the spike protein resulted in an increase in disease severity" => add a citation.

*Reply: In this section in the manuscript (note line numberings have changed) we have made reference to Ilmjarv et al 2021, Volz et al 2021 and Korber et al. 2020 in which no correlation or strong correlation with potential changes in disease severity/mortality was observed with these substitutions compared to the prior ‘Wuhan’ variants.*

The bioproject data is not yet public.

*Reply: We have now made this publicly available.*

Reviewer #2:

Goldswain et al investigated the apparent co-occurrence of P323L in NSP12 and D614G in spike and argued that P323L may play a role in viral transmission. They went on to show perhaps unexpectedly only P323L was preferentially selected in vivo but not D614G despite both being present with a similar proportion in the virus stock used. They then chose to characterise P323 and 323L in vitro on a D614G backbone and concluded that 323L may contribute to enhanced virus transmission. Overall, the genomic analyses seem to be robust with multiple sequencing methods applied and good threshold cut-offs chosen. However, the analysis could be much strengthened by including early collected samples worldwide, especially before wave 1 where only 9 samples were at present analysed. Proper controls are required to support their claims for in vitro studies.

Major points:

It would be useful to provide the frequencies of minor populations in spike and NSP12 from the intra-patient samples before and after the appearance of the double mutants. This could provide some clues for the fast evolution of P323L and D614G (within two days). Depending on its frequency, it may also provide some evidence on why the double mutants, being a minor population, failed to evolve in vivo simultaneously in animal studies. This is important since the authors would like to make a case for monitoring the minor viral population in predicting the outcome of future variants.

*Reply: For the point raised by the reviewer about world-wide frequency we have obtained SARS-CoV-2 sequences and associated sequencing data to derive minor variant from January 2020 – we have also done this for the UK. We have created a new figure and also added to Figure 2.*

*To answer their question with regard to the double positions as e mutants and their frequency we have changed Figure 1A and B to show the two positions as a frequency rather than ratio. We also note that we only had these two samples and no further samples taken on either side.*

The authors wanted to make a case that the double mutants co-occurred despite the animal studies suggesting otherwise. Then they went on to make recombinant viruses based on D614G. The reason for choosing 614G over D614 needs to be justified.

*Reply: We have added this justification in the section ‘The P323L substitution in NSP12 results in a change in plaque phenotype and increased viral RNA and protein synthesis in the context of viruses generated through reverse genetics with G614 in the spike protein’. We have provided the following text ‘The background context of a genome with G614 in spike was chosen because this substitution has been proposed to have occurred first or concurrently with P323L in NSP12 in variants assigned to lineage B. Likewise, there is no evidence of persistence of D614 in the global population of SARS-CoV-2 as part of a dominant sequence.’*

Line 225-231: The authors argued that there was an increase in L across all the animals even post 1 day inoculation and this population became dominant by the end of infection in all animals. However, Figure 4 suggests this is not the case in particular in animals C4 and R4 whereby the dominant was still P on day 5.

*Reply: We have changed the text for this section (Longitudinal analysis of variation in NHPs and cell culture) to reflect the correct observation of the reviewer.*

Line 273: The virus titer needs to be shown; also to provide the type of cells on which the plaque assays were conducted. Figure 6 suggests that despite 323L replicating faster the infectious titer is unaltered post 24 hr of infection. It is plausible that this phenotype is due to the non-productive replication of 323L bearing NSP12 and/or the forming of non-infectious virions in the presence of 323L. This needs to be extended in the discussion. But the experiment addressing the question of whether or not 323L confers a selective advantage would be to infect the cells with mixed P323 and 323L viruses followed by quantitating the virus population over time.

*Reply: We have reported the viral titres for the two viruses created through reverse genetics in the text in the section ‘The P323L substitution in NSP12 results in a change in plaque phenotype and increased viral RNA and protein synthesis in the context of viruses generated through reverse genetics with G614 in the spike protein’. These titres were reported for the two viruses grown in either VeroE6, ACE2-A549 or Vero/hSLAM cells and titred in VeroE6 cells.*

*We disagree with the thoughts of the reviewer about the possible phenotype of L323 being down to the formation of non-productive virions or non-productive replication. If this were the case then no or little virus would be made – and this is not the case? As we show enhanced viral and protein associated with L323 compared to P323. However, the reviewer raises an interesting point about selective advantage. We have added text to the discussion to point out that lineage B accumulated P323L and D614G, whereas lineage A only incorporated D614G.*

*To further explore the idea of the reviewer, we serial passaged the Victoria isolate (Table 1) used in the non-human primate studies. This had both L323 and G614 at very low frequency. The virus was passaged 10 times and at the end of which the frequency of L323 had increased from 0.0003% to 5.4%. we have reported this in the text and presented this data in Figure 6D.*

Line 280: It was assumed 614G conferred more advantage over D614 in viral transmission but this difference was not directly compared in their system. Hence it is incorrect to say the difference in viral RNA/protein is 'marginal' in comparison to the difference seen in the acquisition of D614G.

*Reply: We have removed this statement just to focus on the biology of P323 versus L323.*

Minor points:

The codes for animals should be consistent in Figures 4 and 5. The labels on the X axes should be consistent for Figure 4.

*Reply: We have changed the labels on Figure 4 to match those on Figure 5, as these reflect the original naming convention for the animals, as described in Salguero et al. 2021 (Comparison of rhesus and cynomolgus macaques as an infection model for COVID-19).*

Explain why there are more time points in the same collection for two sequence analyses. For example, animal C3 had 3 collection points for ARTIC but 4 for total RNAseq.

*Reply: This is based on selecting different levels of coverage and this will depend on the sample quality and preparation and sequencing/informatic method. This has been expanded on in the text.*

It is clear that the appearance of 323L is not dependent on the occurrence of D614G in those animals. Is there any evidence to show the appearance of 323L preceded 614G in humans?

*Reply: This is an interesting point and we have discussed this under the context of D614G but not P323L in lineage A variants. We have commented on this in the results.*

Figure 5: There are two types of frequencies as shown in Figure 4. Clarification of which frequencies were used in this analysis and the reasons why they were chosen is needed.

*Reply: We have clarified which frequency is used.*

Figure 6B: It would be useful to show the location of the primer pairs for genomic and subgenomic on the viral genome. Do the error bars represent the technical replicates from one experiment or multiple experiments?

*Reply: Rather than have a diagram of the primer pairs for the genomic and subgenomic RNAs we have referenced this in the methods where we obtained the primer sequence from. The errors bars represent three biological replicates, and this is now stated in the Figure legend with Figure 6.*

Reviewer #3:

This interesting study by Goldswain et.al. convincingly show that the P323L substitution in the SARS-CoV-2 RNA-dependent RNA polymerase (NSP12) gradually dominates the virus population. The strength of the study is the use of powerful sequencing analyses in addition to sound epidemiology. The authors also performed experiments with the hope to bolster the claim that P323L substitution confers growth advantage to the virus. However, this part of the study may be improved by performing a couple of straightforward experiments. My specific comments are as following:

Lines 69-70: The authors wrote " During infection there was rapid selection of 323L into the dominant viral genome sequence but not 614G. In cell culture, recombinant viruses with either P323 or 323L in NSP12 had different phenotypes".

It would be more straightforward if the authors directly describe the actual differences rather than saying "had difference phenotypes".

*Reply: Yes, we have now added text to explain the specific differences – abundance of viral RNA/proteins and a smaller plaque phenotype.*

Lines 120-122: same as above comment.

*Reply: Yes we have now added text, as in answer to the above comment.*

Figure 3: Please describe what the minor variants observed in the N gene are.

*Reply: We have commented in this in the results section.*

Lines 192-194: Again, please explain how the experiment was performed first. MOI, time???

*Reply: We have added details of the route of infection, amount of virus and sampling time points and have added the reference to the original sampling study at further points in this description.*

Line 244-245: The authors stated "323L has a strong selective advantage over P323 in the context of a recombinant virus with the 245 D614 spike variant".

This paragraph needs some expansion to explain how the analysis was performed. There was no description of the recombinant virus. Please provide some experimental details here.

*Reply: We apologise this was our mistake in the original title of this section. This section was not to so with the recombinant virus but was an analysis of the non-human primate data to derive a selection co-efficient. We have clarified this by changing the title and adding further text in the corresponding paragraph to explain the concepts further.*

Lines 226-228: would it be possible for the authors to run PCR and subclone the fragments for Sanger sequencing to prove P323L mutation was already present as a minor genomic variant by Day 1 in some animals?

*Reply: We sequenced the input virus, and show this data in Table 1. The P323L and D614G were present as minor genomic variants – around the 0.003% level, and thus the change was already present.*

Figure 6. Please consider performing additional experiments for panel B and C by including more than one time point if the authors intend to detect any differences in growth kinetics.

*Reply: We did do later time points however, there was cell death and asynchronous infection that would confound analysis. We have added further text to justify why we used the 24 hr time point – mainly around the results of one-step growth curves that show viral release occurs in the 24 hr time window. This allows equivalence across the different reverse genetic viruses.*

**Second round of review**

**Reviewer 2**

The authors have addressed the majority of concerns and the manuscript is much improved. However, the points below need to be addressed before being considered for publication.

Line 197 reference misplaced.

Line 236-238 'We note that whilst this was the general pattern for most individual animals whether cynomolgus or rhesus macaque, there were exceptions including animals C4-1and R2-1.' This statement is slightly misleading. Perhaps it would be more appropriate to directly state the number of animals where L became dominant i.e. 6 out of 12 animals or 50%.

Line 284-286 provide the reference for this statement if it's not concluded from the current study.

Line 289-290 delete 'and Wuhan/G614/P323'

Line 293 those log fold changes seem to be quite big but not sure showing the difference in titre is useful. Suggest providing the titres for both viruses in a table or a bar graph.

Line 420 position 323.

Line 504 described in this chapter?

**Reviewer 3**

My concerns have been addressed.

**Authors Response**

**Point-by-point responses to the reviewers’ comments:**

Reviewer #2:

The authors have addressed the majority of concerns and the manuscript is much improved. However, the points below need to be addressed before being considered for publication.

Line 197 reference misplaced.

*Reply: We have moved the reference inside the brackets.*

Line 236-238 'We note that whilst this was the general pattern for most individual animals whether cynomolgus or rhesus macaque, there were exceptions including animals C4-1and R2-1.' This statement is slightly misleading. Perhaps it would be more appropriate to directly state the number of animals where L became dominant i.e. 6 out of 12 animals or 50%.

*Reply: We have rephrased this to say: “We note that this occurred in 6 out of 12 NHPs with samples sequenced using the ARTIC-Illumina approach and in 5 out of 12 NHPs with samples sequenced using the Illumina total RNAseq (Figure 4).”*

Line 284-286 provide the reference for this statement if it's not concluded from the current study.

*Reply: (Ilmjarv et al., 2021) reference has been added.*

Line 289-290 delete 'and Wuhan/G614/P323'.

*Reply: This has been deleted.*

Line 293 those log fold changes seem to be quite big but not sure showing the difference in titre is useful. Suggest providing the titres for both viruses in a table or a bar graph.

*Reply: A table has been added to Figure 6 as panel 6F to display the average titre (pfu/ml) of three replicates.*

Line 420 position 323.

*Reply: This has been changed to 323.*

Line 504 described in this chapter?

*Reply: This mistake has been rectified.*
